# Macrophages orchestrate elimination of *Shigella* from the intestinal epithelial cell niche via TLR-induced IL-12 and IFN-γ

**DOI:** 10.1016/j.chom.2025.08.001

**Published:** 2025-08-29

**Authors:** Kevin D. Eislmayr, Charlotte A. Nichols, Fitty L. Liu, Sudyut Yuvaraj, Janet Peace Babirye, Justin L. Roncaioli, Jenna Vickery, Gregory M. Barton, Cammie F. Lesser, Russell E. Vance

**Affiliations:** 1 Division of Immunology & Molecular Medicine, Department of Molecular & Cell Biology, University of California, Berkeley, Berkeley, United States; 2 Department of Molecular Biology and Microbiology, Tufts University School of Medicine, Boston, United States; 3 Tufts Stuart B Levy Center for Integrated Management of Antimicrobial Resistance, Tufts University, Boston, Massachusetts, United States of America; 4 Center for Emerging and Neglected Diseases, University of California, Berkeley, Berkeley, United States; 5 Cancer Research Laboratory, University of California, Berkeley, Berkeley, United States; 6 Howard Hughes Medical Institute, University of California, Berkeley, Berkeley, United States

## Abstract

Bacteria of the genus *Shigella* replicate in intestinal epithelial cells and cause shigellosis, a severe diarrheal disease that resolves spontaneously in most healthy individuals. During shigellosis, neutrophils are abundantly recruited to the gut, and have long been thought to be central to *Shigella* control and pathogenesis. However, how shigellosis resolves remains poorly understood due to the longstanding lack of a tractable and physiological animal model. Here, using our newly developed *Nlrc4*^−/−^*Casp11*^−/−^ mouse model of shigellosis, we unexpectedly find no major role for neutrophils in limiting *Shigella* or in disease pathogenesis. Instead, we uncover an essential role for macrophages in the host control of *Shigella*. Macrophages respond to *Shigella* via TLRs to produce IL-12, which then induces IFN-γ, a cytokine that is essential to control *Shigella* replication in intestinal epithelial cells. Collectively, our findings reshape our understanding of the innate immune response to *Shigella*.

## eTOC

Eislmayr et al. reveal a previously unrecognized role of macrophages in controlling the intracellular pathogen *Shigella*. By sensing bacteria and triggering IFN-γ production, macrophages, not neutrophils, help gut epithelial cells fight off infection. This study redefines our understanding of the innate immune response during this severe gastrointestinal infection.

## Graphical abstract

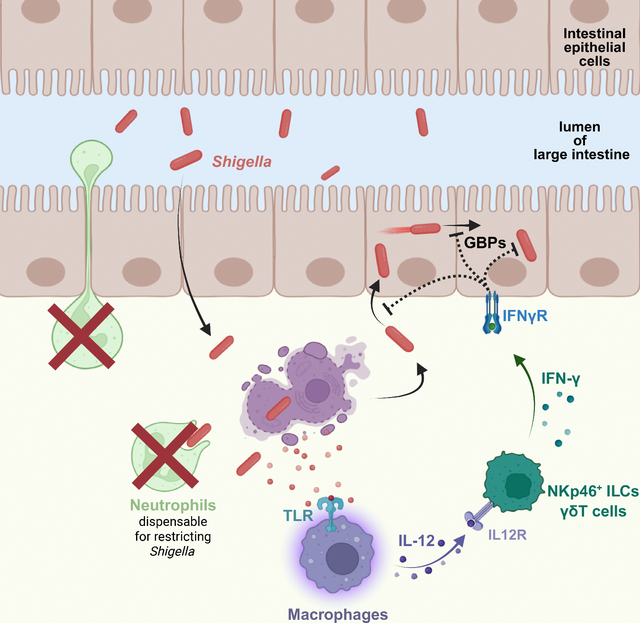

## Introduction

*Shigella* species are the causative agents of shigellosis, a severe inflammatory gastrointestinal infection characterized by symptoms that range from fever, cramps, and nausea to aggressive, mucoid, and bloody diarrhea (dysentery)^1^. Shigellosis is a leading cause of pediatric and geriatric diarrheal-associated mortality, with an estimated 212,000 annual deaths^2–4^. *Shigella* is typically acquired via the fecal-oral route upon ingestion of contaminated food or water^5^. After initial colonization of the gut, *Shigella* is believed to cross the epithelial barrier through M-cells, a specialized epithelial cell-type sampling the gut lumen. *Shigella* then invades intestinal epithelial cells (IECs) by using its Type III Secretion System (T3SS)^6–8^. This needle-like structure enables the cytosolic delivery of ~30 effectors that facilitate bacterial entry into and replication within the cytosol of IECs, the preferred intracellular niche for *Shigella*^7,9^. *Shigella* also uses actin-based motility to spread directly from cell-to-cell within the epithelium^10–12^.

Mice are naturally highly resistant to *Shigella* and are able to resist infectious doses 10,000-fold higher than those sufficient to cause disease in humans^13,14^. Recently, we reported that mice lacking the NAIP–NLRC4 and Caspase-11 inflammasomes are highly susceptible to *Shigella* infection^15,16^. The NAIP–NLRC4 inflammasome detects components of the *Shigella* T3SS^17–19^, and the Caspase-11 inflammasome senses lipopolysaccharide (LPS) of intracellular *Shigella*^20–23^. Susceptibility of *Nlrc4*^−/−^*Casp11*^−/−^ mice to *Shigella* requires pre-treatment of the mice with antibiotics (e.g., streptomycin) in order to facilitate *Shigella* gut colonization, as is also the case for colonization of mice with other gut pathogens^24–26^. Inflammasomes appear to protect mice from *Shigella* via a highly efficient mechanism in which infected intestinal epithelial cells selectively undergo pyroptotic cell death and expulsion into the gut lumen^27,28^. Although human NAIP and NLRC4 are functional and can detect *Shigella*^19^, the human NAIP–NLRC4 inflammasome does not appear sufficient to protect humans, perhaps due to its poor expression in IECs^29^. Additionally, *Shigella* encodes effectors to block host cell death. For example, the *Shigella* effector IpaH7.8 inactivates human—but not mouse—GSDMD, thus preventing pyroptotic cell death^30^. The *Shigella* effector OspC3 potently blocks CASP4 (the human homolog of mouse Caspase-11)^31^, and partially inhibits mouse Caspase-11^23,31,32^. Furthermore, we recently reported that the *Shigella* effector OspF disrupts p38 MAPK-dependent priming of the NAIP–NLRC4 inflammasome, a process essential for rapid inflammasome activation in cells with low basal expression of NAIP–NLRC4^33^. Mice lacking NAIP–NLRC4 and Caspase-11 (*Nlrc4*^−/−^*Casp11*^−/−^) thus recapitulate the lack of inflammasome responses in human IECs, and represent the only genetically tractable animal model that exhibits all the key manifestations of human shigellosis, including oral infection, efficient bacterial replication in IECs, diarrhea that can be bloody, and neutrophilic inflammation^16^.

*Shigella* infection provokes an acute immune response that involves the secretion of proinflammatory factors such as IL-1β and CXCL1, both of which stimulate the recruitment of myeloid cells^34–36^. As a result, extensive infiltration of neutrophils and monocytes is found in biopsies obtained from humans with active shigellosis, and in infected guinea pigs^37–39^. However, the role of myeloid cells during shigellosis remains poorly understood. Surprisingly, loss of IL-1 signaling did not impact the progression of shigellosis in *Nlrc4*^−/−^ mice^16^. Moreover, *Shigella* largely avoids interacting with phagocytes by replicating within and spreading directly within the cytosol of intestinal epithelial cells. Encounters of *Shigella* with macrophages result in the rapid death of macrophages^8,34,40–42^. For all these reasons, macrophages are not believed to be major participants in bacterial control during shigellosis^7^. By contrast, the massive influx of neutrophils to the gut lumen—one of the primary hallmarks of shigellosis—is believed both to promote bacterial clearance and to cause intestinal damage and severe disease symptoms^7,43^. However, direct experimental assessment of the roles of neutrophils and macrophages during infection is lacking. In particular, a possible role for bystander (uninfected) macrophages has not generally been considered, despite the fact these cells are abundant in the gut and would not be subject to *Shigella*-mediated killing.

Here, we demonstrate that *Shigella* infection of *Nlrc4*^−/−^*Casp11*^−/−^ mice exhibits a self-limiting and spontaneously resolving course of disease very similar to that typically seen in human shigellosis. We unexpectedly discover that in this model, neutrophil depletion has no discernable effect on bacterial replication or disease progression, whereas macrophage depletion markedly exacerbates shigellosis symptoms and bacterial burdens in IECs. Our data suggest that macrophages orchestrate the innate response to *Shigella* by initiating a protective cytokine circuit. We propose that this circuit begins with bystander (uninfected) macrophages that detect *Shigella* via Toll-like receptors (TLRs), leading to the production of IL-12. IL-12 stimulates the production of IFN-γ in Nkp46^+^ cells and γδT cells, and we demonstrate IFN-γ is an essential factor in resolving the infection *in vivo* and limiting bacterial replication in human colonic organoids. Mice lacking the ability to produce or sense IFN-γ are incapable of controlling *Shigella* and succumb to the infection. Overall, our study underscores the essential role of pathogen-sensing by macrophages and highlights the pivotal role of IFN-γ in resolving shigellosis *in vivo*.

## Results

**Figure 1:**
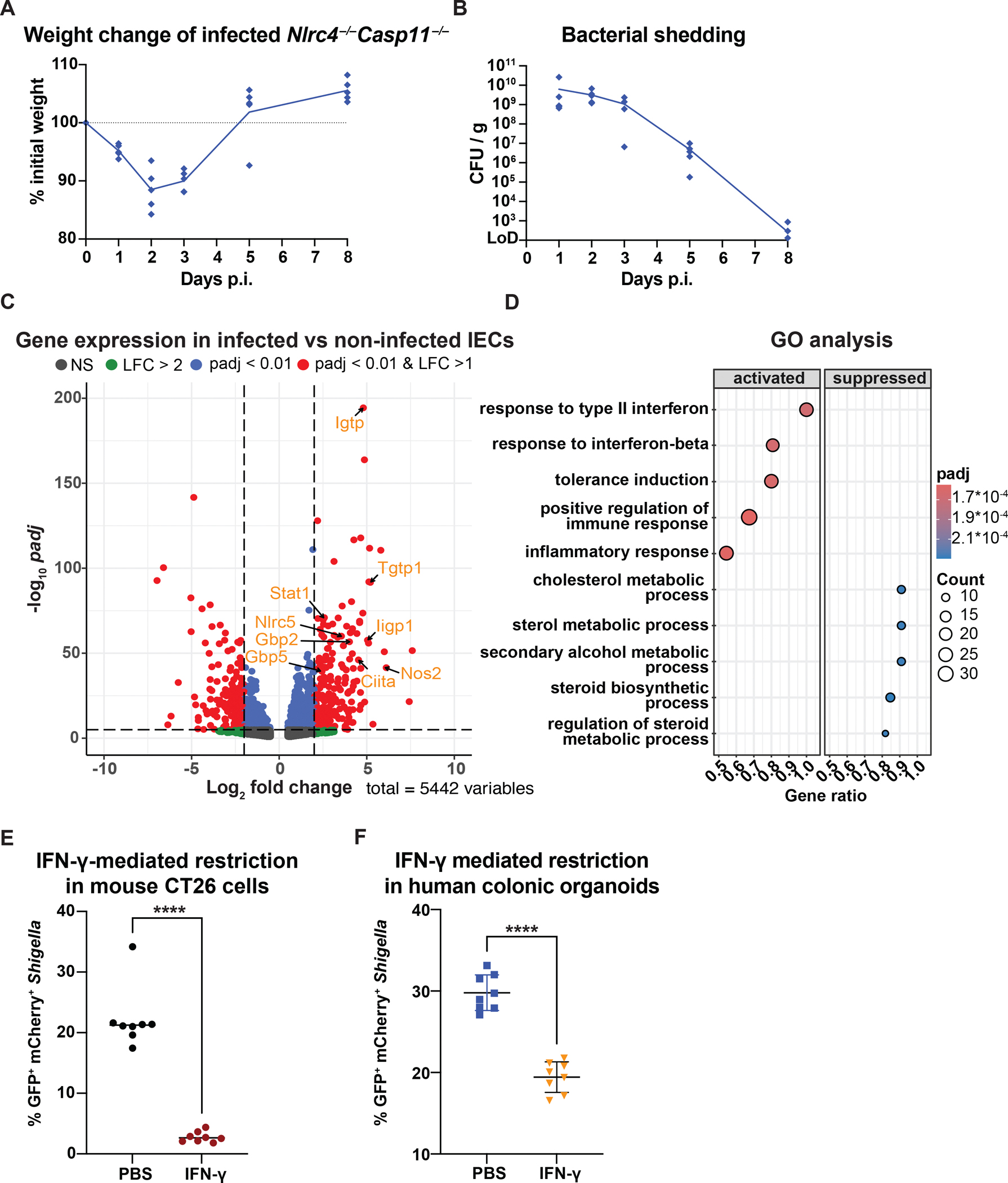
IFN-γ signaling in intestinal epithelial cells is associated with the resolution of *Shigella* infection **(A)** Body weight and **(B)** *Shigella* colony forming units (CFU) in the feces of *Nlrc4*^−/−^*Casp11*^−/−^ mice orally infected with *Shigella flexneri* 2457T. **(C)** Differential expression analysis of bead-enriched EpCAM^+^ colonic-epithelial cells from infected versus non-infected mice (n=6/group). Genes with log_2_ fold change > 1 and padj < 0.001 in red. **(D)** Gene ontology (GO) enrichment analysis of significantly differently expressed genes for activated or suppressed biological processes with a gene set size <150. **(E)** *In vitro* infection of mouse CT26 cells pre-treated with IFN-γ or left untreated for 16h and infected with *Shigella* (MOI=1) that constitutively express mCherry and an arabinose-inducible GFP. After gentamycin treatment and arabinose addition, viable *Shigella* (GFP^+^mCherry^+^) was assessed by flow cytometry 3h later. **(F)** Human colonic organoids were pre-treated with IFN-γ or left untreated for 16 hours and infected with *Shigella*. (A) n=5, (B) LoD=Limit of detection, (E-F) combined data from replicate experiments with n=8, ****p<0.0001 in t-test and median shown.

We previously demonstrated that mice deficient in NLRC4 and Caspase-11 (*Nlrc4*^−/−^*Casp11*^−/−^) are susceptible to oral *Shigella* infection^15,16^. In humans, *Shigella* infection is typically self-limiting and resolves within 5–7 days. To assess whether disease also resolves in *Nlrc4*^−/−^*Casp11*^−/−^ mice, we orally infected streptomycin-pretreated *Nlrc4*^−/−^*Casp11*^−/−^ mice with *Shigella flexneri* strain 2457T and tracked body weight and fecal CFU (Fig. 1A–B). As expected, after 48h of infection, mice lost over 10% of their initial weight and shed over 10^9^ *Shigella* CFU/g feces. However, by 72h, mice began to regain weight, coinciding with a decrease in fecal *Shigella* CFU. By day 5, most mice regained or exceeded their initial weight and stopped shedding bacteria by day 8 post-infection.

To identify the signaling pathways involved in restricting *Shigella* replication, we performed bulk RNA sequencing of bead-enriched EpCAM^+^ epithelial cells isolated from the colon and cecum of mice 48h post-infection. We focused on the cecum and colon since bacteria are almost exclusively detected in IECs from these tissues (Fig. S1A). We found 474 genes were significantly upregulated 48h after the infection when compared to naïve mice (log_2_ fold change (lfc) >2, padj < 0.001, Fig. 1C, Table S1). Among the top differentially expressed genes were *Igtp*, *Tgtp1*, *Nos2* and *Gbp2*, all of which are known to be induced by IFN-γ. Additional interferon-stimulated genes (ISGs) upregulated by infection included *Stat1* and *Ciita* (complete list of canonical ISGs in Table S2). Indeed, gene set enrichment analysis identified “responses to type II interferon” as the top activated biological process (FDR<0.05, Fig. 1D and S1B) for genes with |lfc| > 1, *p*adj <0.05 and a max GO set size <120.

### IFN-γ restricts *Shigella* replication *in vivo* and protects against shigellosis disease

Consistent with previous results demonstrating that IFN-γ inhibits the growth of *Shigella* in mouse embryonic fibroblasts^44^, we found that pretreatment of CT26 cells (a mouse colon carcinoma cell line) with IFN-γ for 16h resulted in substantially fewer cells harboring metabolically active *Shigella* 3h after infection (Fig. 1E, Fig. S1C). Similarly, 16h pretreatment of human colonic organoids with IFN-γ significantly reduced the proportion of *Shigella*-infected cells (Fig. 1F, Fig. S1D).

**Fig. 2:**
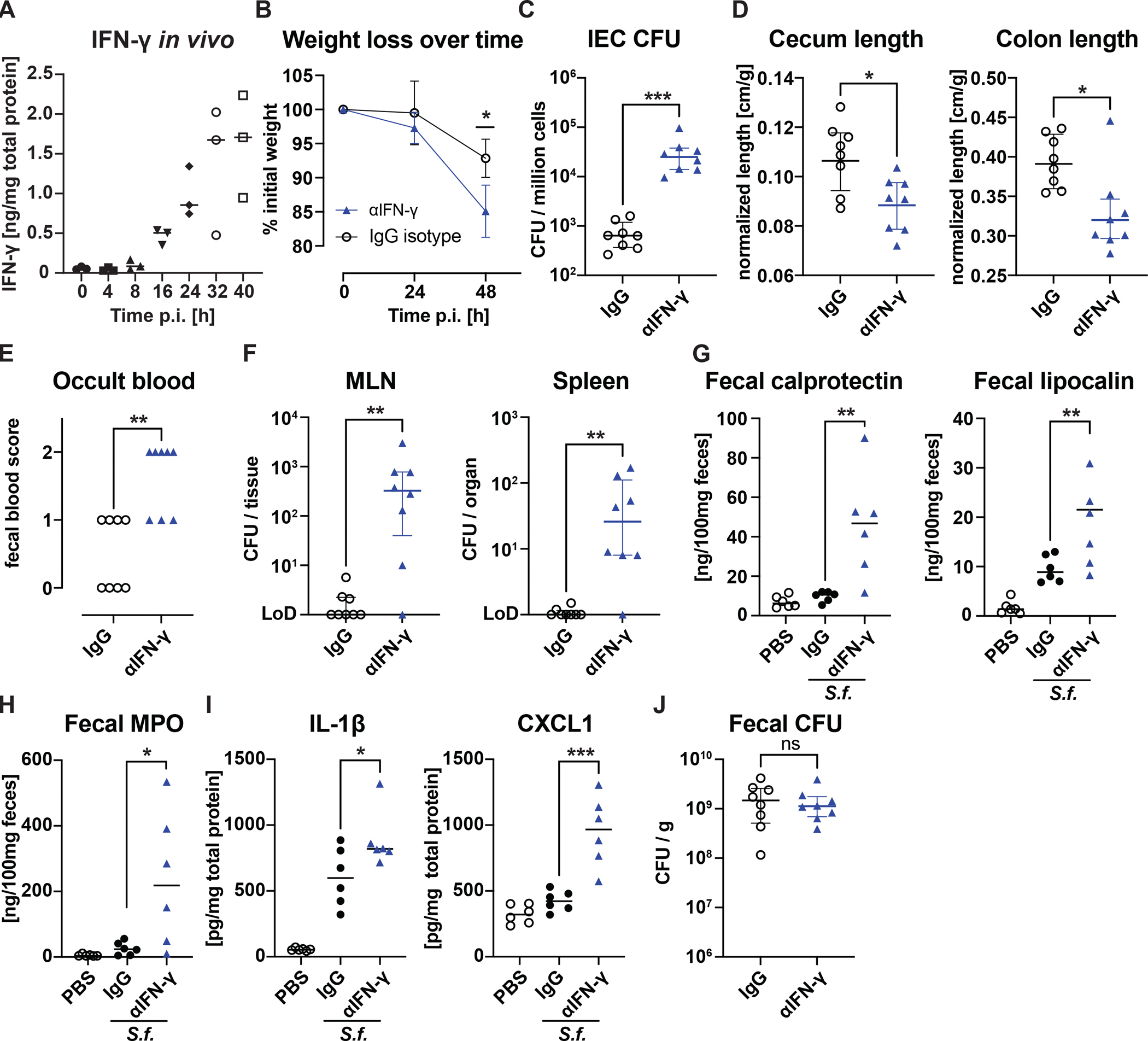
IFN-γ is essential to protect from severe disease and limit bacterial replication **(A)** ELISA quantification of IFN-γ levels in the lamina propria of infected *Nlrc4*^−/−^*Casp11*^−/−^ mice at indicated time points (n=3 per time point). Mice treated with anti-IFN-γ antibody or isotype were assessed for **(B)** body weight, **(C)** intracellular CFU in cecum/colon IECs, **(D)** length of cecum and colon normalized to the initial weight, **(E)** fecal occult blood score (see Methods), **(F)** bacterial dissemination into the mesenteric lymph nodes (MLN) and the spleen, **(G-I)** clinical markers of gut inflammation (calprotectin, lipocalin and MPO in the feces) as well as the pro-inflammatory cytokines IL-1β and CXCL1 levels in lamina propria. PBS-treated non-infected mice steady indicate basal levels. **(J)** Quantification of shed *Shigella* bacteria in the fecal matter of infected animals. Data from two independent experiments with n = 6 PBS and 8 infected mice per treatment group. *p < 0.05, **p < 0.01, ***p < 0.001 in t-test with mean and SD shown (B,D,G,H,I) or Mann-Whitney-test with median and interquartile range (C,E,F,J). ns = not significant (p>0.05).

*In vivo*, we observed IFN-γ levels rise in the lamina as early as 8h after infection, and continue to increase throughout the infection (Fig. 2A). To test whether IFN-γ is important to resolve *Shigella* infection, we treated *Nlrc4*^−/−^*Casp11*^−/−^ mice with an IFN-γ-neutralizing antibody every 24h throughout the infection (starting simultaneously with infection). Anti-IFN-γ-treated mice lost significantly more weight, and harbored >100-fold more *Shigella* CFU in IECs with more pronounced colonic and cecal atrophy—a hallmark of intestinal inflammation—compared to isotype-injected controls (Fig. 2B–D, S2A). Five out of 8 anti-IFN-γ treated mice exhibited high levels of occult blood and the remaining 3 tested faintly positive for occult blood, while only half of the control animals were faintly positive (Fig. 2E). Of note, mice treated with anti-IFN-γ also harbored higher levels of bacteria in their mesenteric lymph nodes and spleens (Fig. 2F), increased fecal calprotectin, lipocalin and myeloperoxidase (MPO, Fig. 2G, H) as well as elevated inflammatory cytokines in the lamina propria (Fig. 2I). Notably, *Shigella* CFU within the feces was similar between anti-IFN-γ and isotype-treated mice, indicating that altered intestinal luminal colonization did not explain the effects of IFN-γ neutralization (Fig. 2J). From these results, we conclude that IFN-γ restricts intracellular *Shigella* replication and prevents severe disease in *Nlrc4*^−/−^*Casp11*^−/−^ mice *in vivo*.

We subsequently evaluated whether neutralization of IFN-γ renders wild-type (*Nlrc4*- and *Casp11*-sufficient) mice susceptible to shigellosis. In undiluted lysates of isolated IECs, we detected only a few (0–5) colonies, regardless of IFN-γ neutralization (Fig. S2B). These low CFU counts are comparable to those observed in mice infected with the virulence plasmid-cured, non-invasive BS103 strain. Therefore, we conclude that *Nlrc4* and *Casp11* dominantly protect the intestinal epithelial cell from *Shigella*, and IFN-γ is essential for protecting that niche if the primary *Nlrc4*/*Casp11*-mediated defense is absent or circumvented.

### IFN-γ is essential for *Shigella* control and acts predominantly on non-hematopoietic cells

**Figure 3.**
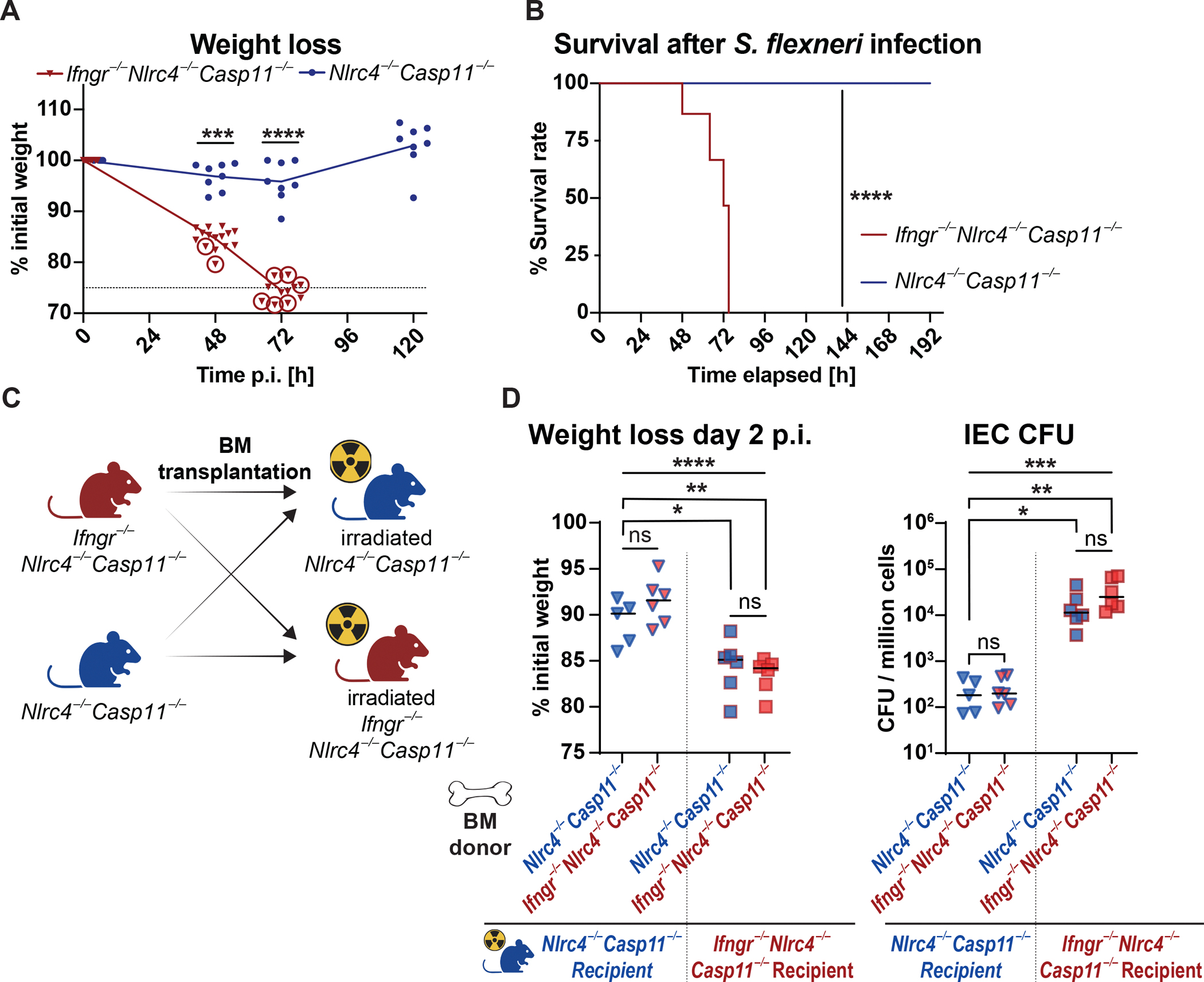
The responsiveness of non-hematopoietic cells to IFN-γ is crucial for controlling *Shigella* infection. **(A)** Change of body weight over time and **(B)** the percentage of mice exceeding humane endpoint criteria (indicated with circled symbols) of infected *Ifngr1*^−/−^*Nlrc4*^−/−^*Casp11*^−/−^ or *Nlrc4*^−/−^*Casp11*^−/−^ mice. The dashed line in (A) represents the predefined 75% weight retention limit set as a humane endpoint. **(C)** Experimental design of reciprocal BM transplantation, created in BioRender. Eislmayr, K. (2025) https://BioRender.com/1dzqp93 **(D)** change in weight and IEC CFU in bone marrow chimeras 48 hours after infection. Donor and irradiated recipient genotypes are as indicated. n=8–15 (A, B) t-test (A) and Log-rank (Mantel-Cox) test (B). n=5–6/group from two replicate experiments in (D) with one-way ANOVA with Tukey’s multiple comparison for weight with median and Kruskal-Wallis test with Dunn’s multiple comparison for CFU. *p<0.05, **p<0.01, ns = not significant (p>0.05).

To provide a genetic test of the role of IFN-γ in *Shigella* control, we generated *Nlrc4*^−/−^*Casp11*^−/−^ mice lacking the IFN-γ-receptor (*Ifngr1*). Consistent with the effects of IFN-γ neutralization (Fig. 2), infected *Ifngr1*^−/−^*Nlrc4*^−/−^*Casp11*^−/−^ mice experienced greater weight loss burdens than IFN-γ-sufficient *Nlrc4*^−/−^*Casp11*^−/−^ animals 48h after infection (Fig. 3A–B). Strikingly, all *Ifngr1*^−/−^*Nlrc4*^−/−^*Casp11*^−/−^ mice reached predefined humane endpoints (e.g., <75% starting body weight, severe signs of morbidity) 72h post-infection, while all *Nlrc4*^−/−^*Casp11*^−/−^ mice survived and regained their initial weight. Notably, anti-IFN-γ treatment of *Ifngr1*^−/−^*Nlrc4*^−/−^*Casp11*^−/−^ mice did not further enhance susceptibility to infection (Fig. S3A). Together, our data indicate a critical role for IFN-γ in restricting *Shigella* and in promoting recovery.

Next, we sought to determine whether IFN-γ acts on hematopoietic or non-hematopoietic cells by generating reciprocal bone marrow chimeras between *Ifngr1*^−/−^*Nlrc4*^−/−^*Casp11*^−/−^and *Nlrc4*^−/−^*Casp11*^−/−^ mice (schematic in Fig. 3C). After 8 weeks of reconstitution, the chimeric mice were infected. We found resistance to *Shigella* depends on the genotype of the bone marrow recipient, regardless of the genotype of the donor (Fig. 3D). Recipients lacking the IFN-γ receptor phenocopied full-body IFN-γ receptor deficiency, regardless of the genotype of the donor bone marrow. Conversely, *Shigella* replication in IECs was restricted in irradiated *Nlrc4*^−/−^*Casp11*^−/−^ mice reconstituted with either bone marrow. From these results, we conclude that IFN-γ predominantly acts on radioresistant (likely non-hematopoietic) cells to restrict *Shigella* replication. Considering the observed profound upregulation of ISGs in IECs during infection (Fig. 1C), our data are consistent with the hypothesis that IFN-γ restricts *Shigella* replication by eliciting an anti-bacterial response in IECs.

Given that type I IFNs and IFN-γ induce a similar set of ISGs, we assessed the role of type-I IFNs during *Shigella* infection. An antibody targeting the Interferon-alpha/beta receptor 1 (αIFNAR) was administered every 24 hours throughout the infection. No significant differences in weight loss or IEC CFU counts were observed between the anti-IFNAR-treated and isotype-treated *Nlrc4*^−/−^*Casp11*^−/−^ or *Ifngr1*^−/−^*Nlrc4*^−/−^*Casp11*^−/−^ animals (Fig. S3B). Thus, type-I IFNs appear to be dispensable in controlling *Shigella* replication in our mouse model, in contrast to the pivotal and essential role of IFN-γ.

*Gbp2* and *Gbp5* were among the top upregulated ISGs in IECs during infection (Fig. 1C). Indeed, GBPs have been implicated in the cell-intrinsic restriction of intracellular pathogens, including *Shigella*^45,46^. However, *Shigella* expresses the effector IpaH9.8, which has been demonstrated to counteract the activity of GBPs^47,48^. Therefore, we next evaluated the role of GBPs in controlling the replication of *Shigella* within CT26 cells. IFN-γ-pretreated *Gbp1/2*^−/−^ and *Gbp5*^−/−^ cells had a significantly higher percentage of GFP^+^ cells than IFN-γ-treated WT cells (Fig. S3C). *Gbp1/2/5*^−/−^ cells were even more susceptible, suggesting that GBPs act combinatorially to limit *Shigella*. Importantly, it is evident that additional ISGs contribute to IFN-γ-mediated restriction, since IFN-γ is still able to reduce *Shigella* burdens in *Gbp1/2/5*^−/−^ (Fig. S3C). Infection of IFN-γ-treated WT cells with an IpaH9.8-deficient *Shigella* strain resulted in a markedly reduced number of infected cells compared to the infection with the WT *Shigella* strain. Notably, in the absence of GBP1/2/5, there was no discernible difference between infections with the WT and IpaH9.8-deficient strain. Collectively, these data indicate that IpaH9.8 partially mitigates the effect exerted by GBPs. In support of this, IpaH9.8-deficient *Shigella* colonized IECs of *Nlrc4*^−/−^*Casp11*^−/−^ mice less efficiently, and caused less weight loss, as compared to WT *Shigella* (Fig. S3D). Moreover, the attenuation of the IpaH9.8 mutant is exclusively observable in the context of an IFN-γ response.

### Macrophages are essential for *Shigella* control, but neutrophils are dispensable

**Figure 4.**
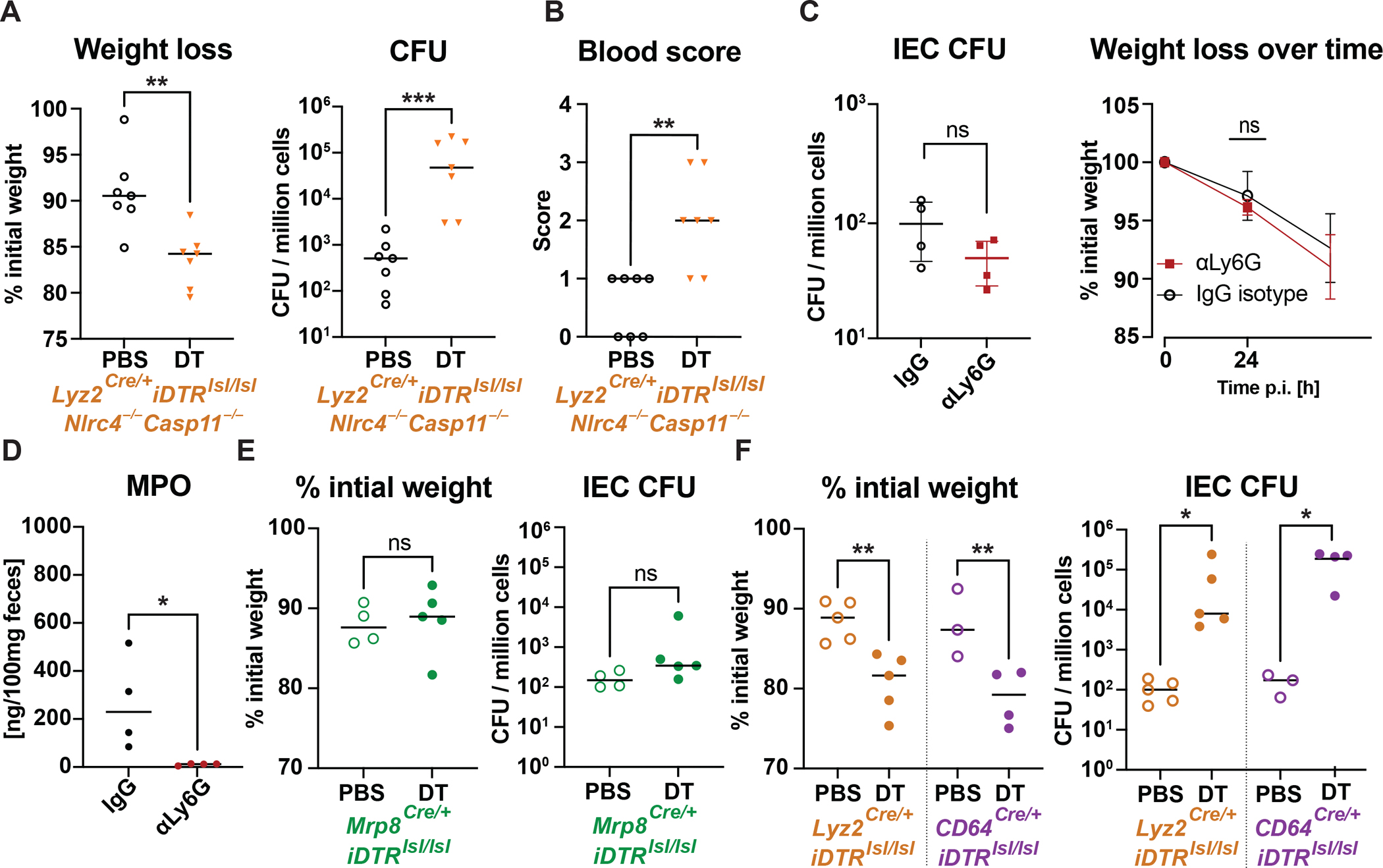
LysM^+^ and CD64^+^ myeloid cells, but not neutrophils, are essential to control shigellosis. **(A)** Change in body weight, IEC CFUs and **(B)** fecal occult blood score of infected *Lyz2*^*Cre/*+^*iDTR*^*lsl/lsl*^*Nlrc4*^−/−^*Casp11*^−/−^ mice in myeloid cell-depleted (DT-treated) or non-depleted (PBS-treated) mice. **(C)** IEC CFU counts, weight loss and **(D)** fecal MPO levels of *Shigella-*infected *Nlrc4*^−/−^*Casp11*^−/−^ mice treated with a neutrophil depleting antibody (αLy6G) or an isotype control. **(E)** Weight loss and IEC CFU counts obtained 48 hours after infection from irradiated *Nlrc4*^−/−^*Casp11*^−/−^ mice reconstituted with *Mrp8*^*Cre/*+^*iDTR*^*lsl/lsl*^ bone marrow and treated with DT (neutrophil depletion), or with PBS. **(F)** Body weight and IEC CFUs of irradiated *Nlrc4*^−/−^*Casp11*^−/−^ mice engrafted with *Lyz2*^*Cre/*+^*iDTR*^*lsl/lsl*^, or *CD64*^*Cre/*+^*iDTR*^*lsl/lsl*^ bone marrow, respectively, treated with DT or PBS (n=4/group). Data from two independent experiments with n = 7 (A-B), n = 4 (C-D), n = 3–5 (E), n = 4–5 (F). *p<0.05, **p<0.01, ***p<0.001, ns = not significant according to t-test (A,D, weight data in E) with mean and SD sown or Mann-Whitney-test (CFU data in A,B,C,E) with median and interquartile range, one-way ANOVA with Tukey’s multiple comparisons for weight (F) and median and Kruskal-Wallis test with Dunn’s multiple comparison for CFU (F).

Although IFN-γ did not act on hematopoietic cells to restrict *Shigella*, we hypothesized that immune cells might still be critical for IFN-γ production. We observed a 15-fold increase in neutrophils (Ly6G^+^Ly6C^med^) and a 5-fold increase in inflammatory monocytes (Ly6G^−^Ly6C^high^MHCII^+^) in the lamina propria during *Shigella* infection (Fig. S4A). To determine if myeloid cells contribute to *Shigella* clearance, we generated *Nlrc4*^−/−^*Casp11*^−/−^ mice with a Cre-inducible diphtheria toxin receptor expressed by LysM^+^ (myeloid) cells (*Lyz2*^*Cre/*+^*iDTR*^*lsl/lsl*^*Nlrc4*^−/−^*Casp11*^−/−^). In these mice, diphtheria toxin (DT) results in the depletion of myeloid cells—including primarily macrophages and neutrophils (Figure S4B–C)—and subsequent *Shigella* infection resulted in a significantly higher weight loss and ~100-fold more CFUs in IECs, as compared to non-depleted mice (Fig. 4A). In addition, myeloid cell depletion also increased infection pathology, resulting in increased inflammatory cytokines (Figure S4D) and enhanced levels of occult/visible blood in the feces (Fig. 4B). Thus, myeloid cells critically contribute to the control of shigellosis.

The recruitment of neutrophils into the gut tissue is believed to play an important role both in controlling bacterial burdens and in causing pathology during shigellosis. Therefore, we assessed the impact of neutrophil depletion, using anti-Ly6G antibody (clone 1A8), during infection. Unexpectedly, we observed that neutrophil depletion did not affect bacterial burdens within IECs, weight loss, or the induction of inflammatory markers during *Shigella* infection, compared to non-treated animals (Fig. 4C and Fig. S4E). Consistent with successful neutrophil depletion (Fig. S4F), 1A8-treated mice exhibited reduced pus and MPO levels in the cecum and colon (Fig. 4D, representative images in Fig. S4H). Treatment with 1A8 had no effect on the luminal levels of *Shigella* (Fig. S4G).

To verify these results, we crossed mice expressing a neutrophil-specific Cre (*Mrp8*^*Cre/*+^)^49^ with *iDTR*^*lsl/lsl*^ mice, and used bone marrow from these mice to reconstitute irradiated *Nlrc4*^−/−^*Casp11*^−/−^ mice. Treatment of the chimeric mice with DT did not alter weight loss or IEC CFU burdens during *Shigella* infection as compared to PBS controls (Fig. 4E). However, DT treatment efficiently depleted neutrophils in the blood and lamina propria of infected mice (Fig. S4I, J). Thus, our results indicate that neutrophil depletion has no major effect on the pathogenesis of *Shigella* infection.

Based on the significant impact of myeloid cell depletion on IEC colonization and the negligible role of neutrophils, we investigated the role of macrophages and monocytes in controlling *Shigella* infection. Scott *et al.* recently described the use of *CD64*^*Cre*^ mice for macrophage-specific Cre expression^50^. Bone marrow from *CD64*^*Cre/*+^*iDTR*^*lsl/lsl*^ mice was transplanted into irradiated *Nlrc4*^−/−^*Casp11*^−/−^ mice. DT-treated (CD64^+^ cell-depleted), infected mice experienced a more pronounced weight loss compared to PBS-treated mice (Fig. 4F). Moreover, CD64^+^ cell-depletion increased *Shigella* IEC CFU burdens similar to LysM^+^ cell-depletion. Notably, the lamina propria of infected and DT-injected *CD64*^*Cre/*+^*iDTR*^*lsl/lsl*^ chimeras exhibited a significant absence of neutrophils in addition to the depletion of CD64^+^ macrophages, in both the lamina propria and blood (Fig. S4I, J), with no effect of DT treatment on blood neutrophils in naïve mice (Fig. S4M). Hence, although CD64^Cre^ is specific for monocytes and macrophages under non-inflamed conditions^50^, it may be expressed by neutrophils during inflammation. In our experiments, however, we rule out an essential role for neutrophils with neutrophil-specific depletion with 1A8 treatment and *Mrp8*^*Cre/*+^*iDTR*^*lsl/lsl*^ mice. In addition, specific dendritic cells depletion (using bone marrow chimeras with *Zbtb46*^*DTR/DTR*^ donors into *Nlrc4*^−/−^*Casp11*^−/−^ recipients) did not affect the bacterial load in IECs to the extent observed in CD64^+^ cell-depleted animals (Fig. S4K,L,N). Therefore, we concluded that CD64^+^ macrophages, and not neutrophils or dendritic cells, play a central role in resistance to *Shigella* infection.

### IFN-γ production is dependent on macrophage TLR sensing.

**Figure 5:**
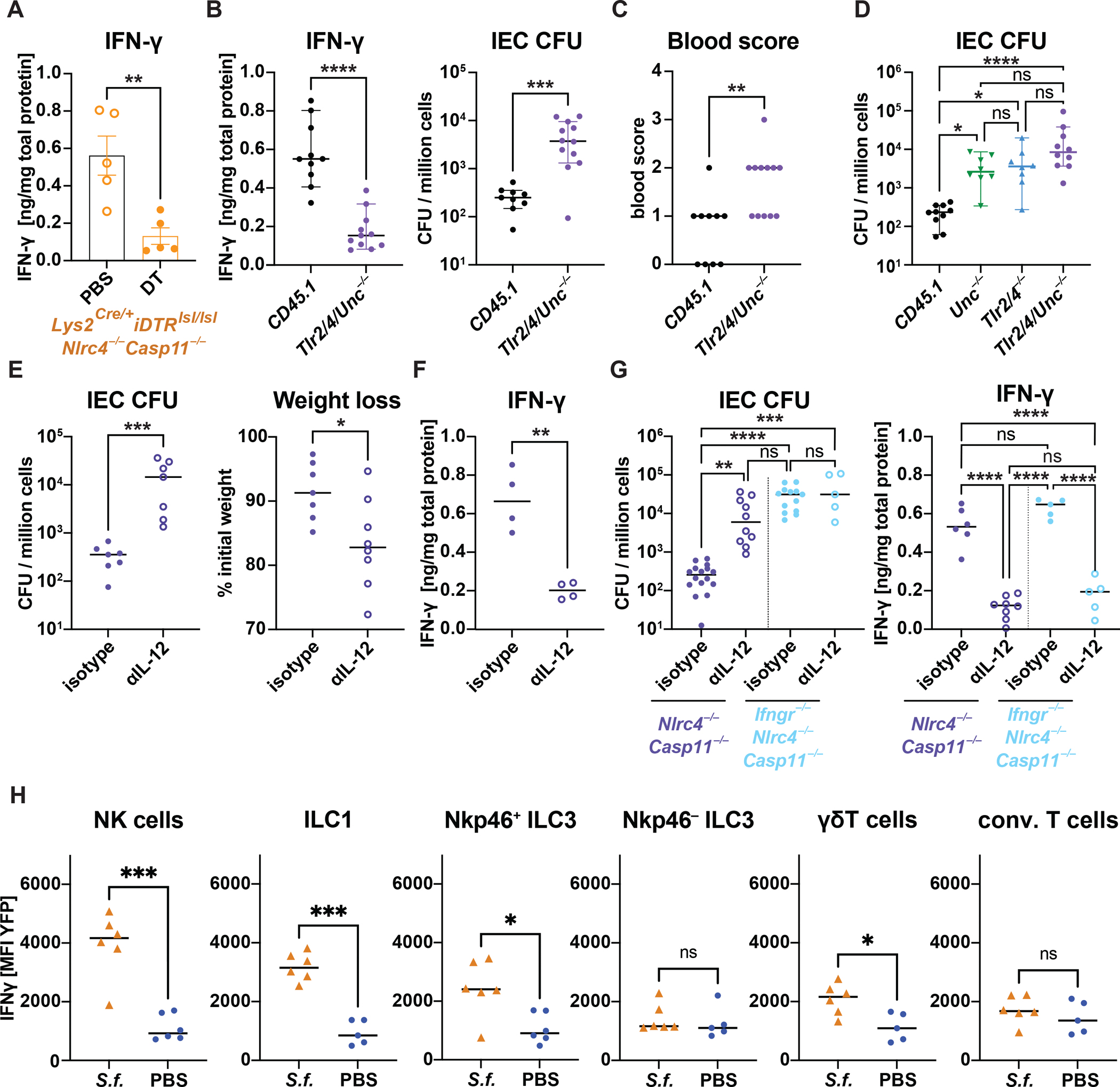
TLR and IL-12 signaling are essential for the production of IFN-γ in Nkp46^+^ and γδT cells to control *Shigella* infection. **(A)** IFN-γ levels in the lamina propria (LAP) of infected *Lyz2*^*Cre/*+^*iDTR*^*lsl/lsl*^*Nlrc4*^−/−^*Casp11*^−/−^ mice with or without DT-induced myeloid cell-depletion. **(B)** IFN-γ in the LAP and IEC CFU 48 hours post-infection in irradiated *Nlrc4*^−/−^*Casp11*^−/−^ bone marrow chimeras receiving *Tlr2/4-Unc*^−/−^ or CD45.1-WT bone marrow and **(C)** the corresponding fecal occult blood score. **(D)** IEC CFU from irradiated *Nlrc4*^−/−^*Casp11*^−/−^ engrafted with either *Unc*^−/−^*, Tlr2/4*^−/−^*, Tlr2/4-Unc*^−/−^ or CD45.1-WT bone marrow. **(E)** Effect of IL-12p40 neutralization (αIL-12) or isotype antibody treatment during *Shigella* infection of *Nlrc4*^−/−^*Casp11*^−/−^ mice on IEC CFU counts, weight loss and **(F)** IFN-γ levels in LAP. **(G)** IEC CFU counts and IFN-γ levels in the LAP of αIL-12 or isotype treated *Nlrc4*^−/−^*Casp11*^−/−^ or *Ifngr*^−/−^*Nlrc4*^−/−^*Casp11*^−/−^ mice. **(H)** Mean fluorescent intensity (MFI) of the GREAT-IFN-γ-YFP reporter in different cell-types isolated from the LAP of *Shigella*-infected (*S.f*.) or mock-treated (PBS) animals that had been irradiated *Nlrc4*^−/−^*Casp11*^−/−^ and received bone marrow from an IFN-γ reporter strain. Mean and t-test (A,F,H), median and Mann-Whitney-test (B CFU, C, F CFU and weight loss), median and Dunn’s multiple comparisons test (CFU data in G) and mean and Tukey’s multiple comparisons test (IFN-γ data in G). Combined data from replicate experiments with n=5 (A), n=9–12 (B), n=7 (F,G), n=8–10 (D), n=5–16 (G).

Since both IFN-γ neutralization and depletion of macrophages/monocytes led to heightened bacterial IEC colonization, we wondered whether macrophages/monocytes might play a role in IFN-γ induction during infection. Indeed, IFN-γ levels were significantly reduced in the lamina propria upon myeloid cell-depletion (Fig. 5A).

Macrophages sense bacterial infections via Toll-like receptors (TLRs) and produce pro-inflammatory cytokines, including the IFN-γ-inducing cytokine, IL-12. It has been demonstrated that *Unc93b1*-deficient cells no longer respond to TLR3, TLR7, TLR9, TLR11, TLR12, and TLR13 ligands^51^. By crossing these mice to *Tlr2*^−/−^ and *Tlr4*^−/−^ mice, Sivick et al generated mice incapable of TLR signaling^52^, while still retaining the capacity for IL-1R and IL-18R signaling. Bone marrow chimeras in which *Nlrc4*^−/−^*Casp11*^−/−^ recipients were reconstituted with *Tlr2/4/Unc*^−/−^ donor marrow exhibited blunted IFN-γ induction compared to WT(CD45.1) into *Nlrc4*^−/−^*Casp11*^−/−^ control chimeras (Fig. 5B). Moreover, loss of TLR signaling by hematopoietic cells also resulted in elevated IEC CFU and enhanced pathology (Fig. 5C and S5A). Of note, the IEC CFU counts in either *Tlr2/4*^−/−^ *or Unc*^−/−^ bone marrow chimeras were intermediate between those observed in CD45.1 or *Tlr2/4/Unc*^−/−^ engrafted *Nlrc4*^−/−^*Casp11*^−/−^ animals (Fig. 5D). Consequently, the restriction of *Shigella* appears to depend on a combinatorial Unc-dependent and Unc-independent TLRs response.

To determine whether TLRs function within macrophages to orchestrate the protective response to *Shigella*, we generated bone marrow chimeras in which *Nlrc4*^−/−^*Casp11*^−/−^ mice were reconstituted with a mix of *CD64*^*Cre/*+^ *iDTR*^*lsl/lsl*^ and *Tlr2/4/Unc*^−/−^ donor bone marrow. In these chimeras, half of the bone marrow-derived CD64^+^ macrophages are DT-sensitive, while the other half lacks TLR signaling. Upon DT-treatment, only CD64^−^ cells in these mice will express functional TLRs. Consistent with a critical role for TLRs in CD64^+^ cells, DT-treatment of the mixed chimeras elevated the bacterial load within IECs to levels comparable to those of 100% *CD64*^*Cre/*+^ *iDTR*^*lsl/lsl*^ or 100% *Tlr2/4/Unc*^−/−^ chimeras, and similar to *Ifngr1*^−/−^*Nlrc4*^−/−^*Casp11*^−/−^ mice (Fig. S5B). Notably, without DT-treatment, the mixed chimeras exhibited IEC CFU counts comparable to non-irradiated *Nlrc4*^−/−^*Casp11*^−/−^ mice. Collectively, our data demonstrate the significant role of TLR-mediated sensing in CD64^+^ macrophages to initiate the protective IFN-γ response to *Shigella*.

### Macrophage-derived IL-12 induces IFN-γ production in Nkp46^+^ and γδT cells

IFN-γ can be induced by different cytokines, including IL-12, IL-15, IL-18, and type I IFN^53,54^. Interperitoneal injection of IL-18 neutralizing antibody during *Shigella* infection of *Nlrc4*^−/−^*Casp11*^−/−^ mice had no effect on IEC CFU burdens or pathology (Fig. S5C). By contrast, IL-12p40 neutralizing antibody significantly increased IEC CFU burdens (Fig. 5E), exacerbated weight loss (Fig. S5D), and aggravated the inflammatory symptoms of shigellosis (Fig. S5E–G), as compared to isotype control. Notably, IL-12p40 neutralization resulted in a significant reduction of IFN-γ in the lamina propria (Fig. 5F), and produced symptoms comparable to *Ifngr1*^−/−^*Nlrc4*^−/−^*Casp11*^−/−^ mice. Moreover, treatment with anti-IL-12p40 had no effect on the severity of *Shigella* infection in the absence of IFN-γR (Fig. 5G, S5D–G), consistent with a model in which the main role of IL-12 is to mediate resistance to *Shigella* via induction of IFN-γ.

Next, we examined whether macrophages are the source of IL-12 in our infection model. As ELISA or flow cytometry-based assays were not sensitive enough, we generated mixed bone marrow chimeras using *CD64*^*Cre/*+^ *iDTR*^*lsl/lsl*^ and *Il12b*^−/−^ donor mice. In these chimeras, half of the hematopoietic cells are incapable of producing IL-12p40 (due to *Il12b* deficiency), but any CD64^+^ cells able to produce IL-12 are sensitive to DT-depletion. Thus, upon DT treatment, IL-12p40 can only originate from CD64^−^ cells. Without DT, mixed chimeras exhibited IEC CFU counts akin to those of *Nlrc4*^−/−^*Casp11*^−/−^ mice (Fig S5H). In stark contrast, DT treatment resulted in a 100-fold increase in the IEC bacterial loads, and additional administration of either IL-12p40 or IFN-γ neutralizing antibodies caused only a modest and insignificant additional elevation. The elevated bacterial counts were comparable to DT-treated CD64^Cre/+^ iDTR^lsl/lsl^*Nlrc4*^−/−^*Casp11*^−/−^ bone marrow chimeras or irradiated *Nlrc4*^−/−^*Casp11*^−/−^ mice receiving *Il12b*^−/−^ bone marrow. We therefore infer that CD64^+^ cells are a critical source of IL-12p40 during *Shigella* infection. In an analogous set of experiments, we generated bone marrow chimeras in which irradiated *Nlrc4*^−/−^*Casp11*^−/−^ were engrafted with either a mix of *Tlr2/4/Unc*^−/−^ and *Il12b*^−/−^ bone marrow, or a mix of *CD45.1* and *Il12b*^−/−^ bone marrow as a control. In the *Tlr2/4/Unc*^−/−^:*Il12b*^−/−^ mixed chimeras, cells with functional TLR signaling cannot produce IL-12p40, whereas cells that can produce functional IL-12p40 cannot sense *Shigella* via TLRs. Thus, despite the presence of both TLR-responsive or IL-12p40-producing cells, there are no cells that can both detect *Shigella* via TLRs and produce IL-12p40. Infection of these chimeras with *Shigella* resulted in much higher IEC CFU burdens as compared to the control *Tlr2/4/Unc*^−/−^:CD45.1 chimeras, indicating that the production of IL-12p40 depends on active TLR signaling within the same cell (Fig. S5I). Collectively, our data indicate that TLR sensing in CD64^+^ macrophages induces IL-12 in a cell-autonomous manner, which is essential for orchestrating the IFN-γ-mediated restriction of *Shigella*.

Finally, to identify the IFN-γ-producing immune cells in our infection model, we utilized the GREAT IFN-γ reporter mice^55^. These transgenic mice carry a bicistronic IFN-γ-IRES-eYFP construct under the control of the endogenous *Ifng* promoter and poly(A) tail, with unaltered induction and expression levels of IFN-γ^56^. *Shigella* infection induced an increase in the mean fluorescence intensity (MFI) of YFP within various different immune cell populations in the colon of *Nlrc4*^−/−^*Casp11*^−/−^ animals engrafted with bone marrow from GREAT mice (Fig. 5H; gating in Fig. S5J). We identified NK cells (Lin^−^Rorγt^−^Nkp46^+^Eomes^+^), ILC1 (Lin^−^Rorγt^−^Nkp46^+^,Eomes^−^), Nkp46^+^ILC3 (Lin^−^Rorγt^+^Nkp46^+^) and γδT cells (Lin^+^γδTCR^+^) as the cell-types producing IFN-γ. In contrast, IFN-γ induction was not detected in conventional T cells (Lin^+^TCRβ^+^γδTCR^−^) or other Lineage^+^TCRβ^−^γδTCR^−^ cells (Fig. 5H, representative histogram in Fig. S5K). Based on these findings, we conclude that NK cells, Nkp46^+^ ILCs, and γδT cells are likely responsible for providing the protective IFN-γ in our mouse model of shigellosis.

## Discussion

Bacillary dysentery is a self-limiting disease in most healthy adults. However, the lack of suitable animal models has hindered our ability to determine the key processes that control *Shigella* replication and disease symptoms *in vivo*. We previously showed that *Shigella* successfully invades and replicates in epithelial cells of mice lacking the NLRC4 inflammasome^14,15^. Mice deficient in both NLRC4 and Caspase-11 are even more susceptible to *Shigella*, and exhibit all pathological aspects of human shigellosis^16^. Here, we show that infected *Nlrc4*^−/−^*Casp11*^−/−^ mice recover from dysentery within five days, similar to the typical disease course in humans (Fig. 1A,B).

The swift recovery of infected mice within a few days indicates that the processes that ultimately curtail *Shigella* growth must already be activated in the early stages of the disease, consistent with a pivotal role of the innate immune response. Furthermore, as epithelial cells of the large intestine are the predominant niche for *Shigella*, these innate responses must ultimately restrict or prevent intracellular bacterial replication in IECs. The top differentially expressed genes in IECs after *in vivo* infection are characteristic of an IFN-γ response (Fig. 1C, D, S1B). Indeed, mice failing to mount a response to IFN-γ are incapable of recovering from a *Shigella* infection (Fig. 3A, B) and exhibit severe diarrhea, exacerbated inflammation, and 1,000 times higher CFUs within IECs compared to infected control animals (Fig. 2B–I, S2A, S3A).

Using reciprocal bone marrow chimeras, we found that IFN-γ exerts its effects directly on radioresistant (non-hematopoietic) cells (Fig. 3D). Given that *Shigella* replicates within IECs, the most parsimonious explanation for our data is that IFN-γ is acting directly on infected IECs. Notably, in WT B6 mice, the rapid NAIP–NLRC4-mediated expulsion of infected epithelial cells obviates the need for IFN-γ (Fig. S2B), in line with reports from our lab and others^15,27,57,58^. Notably, it has been previously reported that in human IECs, the NAIP–NLRC4 inflammasome is incapable of mounting a protective response against *Shigella*^29,33^. Therefore, we postulate that IFN-γ is likely to play an important role in humans. This hypothesis is underscored by the restrictive effect of IFN-γ pretreatment on the intracellular replication of *Shigella* in human colonic organoids (Fig. S1D). In addition, more indirect mechanisms of IFN-γ-mediated protection are also possible. Ultimately, specific tissue-specific deletion of *Ifngr1* from IECs (on an *Nlrc4*^−/−^*Casp11*^−/−^ background) will be necessary to confirm whether IFN-γ signaling in IECs is necessary for *Shigella* control *in vivo*.

IFN-γ has been identified as a key factor in restricting several intracellular pathogens, including *Mycobacterium tuberculosis*, *Listeria monocytogenes*, and *Legionella pneumophila*^59–65^, and previous studies have implicated a potential protective activity against *Shigella in vitro* and in a non-physiological intranasal infection model^66–72^. IFN-γ induces the expression of hundreds of interferon-stimulated genes (ISGs) to promote pathogen clearance. Several ISGs have been shown to confer antibacterial functions during *Shigella* infection *in vitro*^45,68,70,73,74^. The most prominent example is the family of guanylate-binding proteins (GBPs). GBPs bind and liberate LPS to promote CASP11- and CASP4-mediated pyroptotic cell death^45,46,48,66,69^. In addition, the binding of GBPs to LPS forms a cage-like structure, trapping intracellular bacteria and inhibiting actin-dependent motility^75,76^. However, *Shigella* disrupts the GBP coat by secretion of an E3 ubiquitin ligase effector protein, IpaH9.8, which ubiquitylates GBPs and targets them for proteasome-dependent degradation^48,68,76^. Our findings underscore the dynamic interplay between *Shigella* virulence factors and host immune defenses. Specifically, the reduced responsiveness to IFN-γ in GBP-deficient CT26 cells indicates a role for GBPs in mediating the protective effects of IFN-γ (Fig. S3C). However, this host defense mechanism is partially subverted by the *Shigella* effector IpaH9.8, as evidenced by the attenuated virulence of IpaH9.8-deficient bacteria *in vitro* and *in vivo* (Fig. S3C, D). In line with previous reports, our results suggest that IpaH9.8 antagonizes GBP-dependent immunity. Of note, it is also evident from our data that additional ISGs contribute to the IFN-γ-mediated restriction, as *Gbp1/2/5* deficiency only partially abolishes the IFN-γ-mediated protection in CT26 cells (Fig. S3C). Indeed, other ISGs, such as Viperin, Apolipoprotein 3 (APOL3), and the E3 ligase RNF213, have been suggested to restrict intracellular *Shigella* to some degree^70,71,73,77^. However, *Shigella* expresses effector proteins that counteract these activities^78^. For instance, IpaH1.4 directly antagonizes RNF213 by mediating its proteasomal degradation^71,77^. Despite *Shigella* antagonism of ISGs, our study in mice identified IFN-γ responses as central in restricting *Shigella in vivo* and in human colonic organoids, with a partial contribution of GBPs.

Despite the widely held belief that neutrophil recruitment and transepithelial migration into the gut lumen are significant drivers of pathology during shigellosis^1,7^, we find no major role for neutrophils during *Shigella* infection *in vivo* (Fig. 4C, D, F, Fig. S4D, E). Importantly, we observed extensive recruitment of neutrophils into the lamina propria (Fig. S4A), and the presence of the neutrophil-specific enzyme myeloperoxidase in the feces. These observations suggest that neutrophils are recruited to the intestine in our model, and cross the epithelial layer, as in humans, in which the presence of neutrophils in the stool is a clinical marker for shigellosis^1,79,80^. Nonetheless, the only noticeable effects of neutrophil depletion were the absence of pus (Fig. S4F) and undetectable MPO levels in the gut lumen, without any impact on weight loss or the bowel inflammation markers lipocalin and calprotectin (Fig. 4C, D, F and S4D, E). Importantly, neutrophil depletion during infection did not affect IEC bacterial counts. It is intriguing that neutrophils appear neither to exacerbate disease nor restrict the growth of *Shigella*. While numerous studies highlight the critical role of neutrophils in eliminating invading microbes and resolving inflammation (as reviewed by Fournier *et al.*^81^), in the case of *Shigella* infection, neutrophils seem to have a negligible impact on bacterial restriction. This might be explained by the ability of *Shigella* to effectively induce cell death of phagocytic cells^7,79^ or by *Shigella* evasion of phagocytes by replication in epithelial cells. Alternatively, neutrophils may enhance bacterial clearance, but this anti-bacterial function is offset by a distinct pro-bacterial (e.g., immunoregulatory) effect of neutrophils. It may also be the case that neutrophils play important roles in preventing systemic spread of *Shigella*, or in the late stages of the infection, neither of which were examined in our study.

*Shigella* rapidly induces macrophage cell death at early stages of infection^43,82,83^. It is generally accepted that *Shigella* induces macrophage death to enable the subsequent infection of epithelial cells^7,8^. Rapid induction of macrophage death is also believed to limit the ability of macrophages to restrict bacterial replication. However, a recent study using a zebrafish model of *Shigella* infection and experiments with autophagy-deficient (*Atg16l1*^−/−^) bone marrow-derived macrophages found a host-protective role for macrophages^84,85^. Thus, it remains unclear whether *Shigella*-mediated macrophage cell death is beneficial to the pathogen or to the host and whether macrophages participate in host defense against *Shigella* intestinal infections. In experiments with mice lacking monocytes and macrophages, we detected increased disease severity and colonization of the intestinal epithelium (Fig. 4A, B, E, and S4C). We also noted similar pathology in mice deficient in TLR signaling (Fig. 5B, C). TLRs sense extracellular-derived microbial ligands, and since *Shigella* is a pathogen that replicates in the cytosol, it is generally believed that cytosolic sensing pathways (e.g., NOD1/2^86^ and ALPK1^87^) are more important in infected cells than TLRs in initiating innate immunity to *Shigella*. However, it is likely that microbial ligands are released extracellularly during infection, and thus, bystander (uninfected) macrophages may sense and respond to *Shigella*. In accord with the results of our *Tlr2/4/Unc*^−/−^ and *CD64*^*Cre/*+^ *iDTR*^*fl/fl*^ mixed bone marrow experiment (Fig. S5B), we propose a model in which the release of bacterial products from infected, pyroptotic macrophages facilitates the release of protective cytokines from noninfected bystander cells—most likely macrophages—in a TLR-dependent manner. Together, our data indicate a beneficial role for macrophages in host defense during *Shigella* infection.

Finally, our results demonstrate that macrophage activation is pivotal in the induction of adequate IFN-γ to restrict *Shigella* (Fig. 5A). Although some reports suggest that macrophages can directly produce IFN-γ, our experiments using IL-12p40 neutralizing antibodies suggest that the secretion of IFN-γ is indirect and reliant on IL-12p40 (Fig. 5G), a cytokine subunit known to be induced in macrophages downstream of TLR activation. Our mixed *Tlr2/4/Unc*^−/−^:*Il12b*^−/−^ bone marrow chimera experiments further suggest that TLR signaling and IL-12p40 secretion occur within the same cell (Fig. S5I). IL-12 is a heterodimeric cytokine composed of the IL-12p35 and IL-12p40 (encoded by *Il12b*) subunits, the latter of which also heterodimerizes with IL-23p19 to form IL-23^88^. Given this shared subunit, we cannot exclude the possibility that IL-23 contributes to the effects of IL-12p40 in our model. However, the functional profiles of IL-12 and IL-23 diverge significantly: IL-12 is classically associated with the induction of T_H_1 and NK cell responses and robust IFN-γ production, while IL-23 predominantly promotes the expansion and stabilization of T_H_17 cells, leading to secretion of IL-17, IL-21, IL-22, and GM-CSF^89–95^. Given that we observe a critical role for IL-12p40 in IFN-γ induction, rather than in type 17 responses, our data suggest that IL-12, rather than IL-23, is the more likely driver of the immune response observed in our system. Additionally, using the transgenic GREAT IFN-γ reporter chimeras, we identified Nkp46^+^ ILCs and NK cells, along with γδ T cells, as potential sources of IFN-γ (Fig. 5H).

In sum, our results necessitate revisions to the generally accepted model of *Shigella* pathogenesis. In particular, our results identify a critical function for macrophages, but not neutrophils, in host defense against *Shigella*. Given that infected macrophages are rapidly killed by *Shigella*, we propose that bystander (uninfected) macrophages are vital in orchestrating anti-*Shigella* innate immunity. Instead of directly killing *Shigella* by phagocytosis, we propose that a TLR–IL-12 circuit induces the expression of IFN-γ, which then acts on IECs to restrict *Shigella* replication. Ultimately, understanding how host immunity coordinates clearance of *Shigella* may be essential for the development of an effective *Shigella* vaccine.

## Resource availability:

### Lead contact

Further information and requests for resources and reagents should be directed to and will be fulfilled by the lead contact, Russell Vance (rvance@berkeley.edu).

### Materials availability

Materials used in this study will be provided upon request and available upon publication.

### Data and code availability

Raw bulk RNA-sequencing data are deposited in the NCBI Gene Expression Omnibus: GSE288567.

## Star Methods

### EXPERIMENTAL MODEL AND STUDY PARTICIPANT DETAILS

Mice were maintained under specific pathogen-free conditions and housed with a 12-hour light-dark cycle and standard chow diet (Harlan irradiated laboratory animal diet) ad libitum in accorsssdance with the regulatory standards of the University of California Berkeley Institutional Animal Care and Use Committee. All mice were sex- and age-matched and were 6–12 weeks old when infected (except mice receiving BM transplantation, see details below). Both male and female mice were used in all experiments. Littermate controls were used or, if not possible, mice were co-housed for at least three weeks prior to infection.

C57BL/6J-*Nlrc4*^542stop^*Casp4*^em13JLR^/J (*Nlrc4*^−/−^*Casp11*^−/−^) animals were previously generated and described by Roncaioli et *al*.^16^ via targeted CRISPR-Cas9 mutagenesis of *Casp11* in existing *Nlrc4*^−/−^ (C57BL/6J-*Nlrc4*^542stop^/J)^96^ mice. 129P2-*Lyz2*^tm1(cre)Ifo^/J (*Lyz2*^*Cre/*+^)^97^, B6.Cg-Tg^(S100A8-cre,-EGFP)1Ilw^/J (*Mrp8*^*Cre*^)^49^, C57BL/6-Gt(ROSA)26Sor^tm1(HBEGF)Awai^/J (*iDTR*^*lsl/lsl*^)^98^, B6.129S7-*Ifngr1*^tm1Agt^/J (*Ifngr1*^−/−^)^99^, B6.129S1-*Il12*^btm1Jm^/J^100^, B6(Cg)-*Zbtb46*^tm1(HBEGF)Mnz^/J^101^, B6.129S4-*Ifng*^tm3.1Lky^/J^55^ mice were purchased from Jackson Laboratories. The *Ifngr1*^−/−^*Nlrc4*^−/−^*Casp11*^−/−^ mouse line was generated by mating *Ifngr1*^−/−^ and *Nlrc4*^−/−^*Casp11*^−/−^ mice. B6-*Fcgr1*^tm2Ciphe^ (*CD64*^*Cre/*+^)^50^ mice were generated by Bernard Malissen at Centre d’Immunologie de Marseille-Luminy and provided by Yasmine Belkaid at the National Institutes of Health. *iDTR*^*lsl/lsl*^*Nlrc4*^−/−^*Casp11*^−/−^ were generated by crossing *iDTR*^*lsl/lsl*^ mice, obtained from the Jackson Laboratory, with our *Nlrc4*^−/−^*Casp11*^−/−^ mice. Subsequently, *Lyz2*^*Cre/*+^*iDTR*^*lsl/lsl*^*Nlrc4*^−/−^*Casp11*^−/−^, *CD64*^*Cre/*+^*iDTR*^*lsl/lsl*^*Nlrc4*^−/−^*Casp11*^−/−^ and *Mrp8*^*Cre/*+^ *iDTR*^*lsl/lsl*^ *Nlrc4*^−/−^*Casp11*^−/−^ lines were generated by crossing *iDTR*^*lsl/lsl*^ *Nlrc4*^−/−^*Casp11*^−/−^ mice to animals form the *Lyz2*^*Cre/*+^, *CD64*^*Cre/*+^ or *Mrp8*^*Cre/*+^ line, respectively. C57BL/6N-*Unc93b1*^*tm1(KOMP)Vlcg*^/Mmucd (*Unc93b1*^−/−^) mice were obtained from the Mutant Mouse Resource and Research Center (MMRRC) at the University of California, Davis, and originally donated to the MMRRC by David Valenzuela of Regeneron Pharmaceuticals^102^ and after crossing to *Tlr2*^−/−^*Tlr4*^−/−^ mice, *Tlr2*^−/−^*Tlr4*^−/−^*Unc*^−/−^ animals were generously provided by Gregory Barton at the University of California, Berkeley.

#### *Shigella* cultivation and preparation for *in vivo* infections.

If not stated otherwise, infection experiments were conducted with a natural streptomycin-resistant strain of *Shigella flexneri* serovar 2a 2457T^32^ (*S.f.*). *S.f.* was grown on tryptic soy broth (TSB, BD Bacto # DF0370–07-5) agar plates containing 0.01% congo red (CR, Sigma-Aldrich # C6767) and 100 μg/mL streptomycin sulfate at 37°C. For infections, a single CR-positive colony was picked from a streak not older than a week, inoculated into 5 mL TSB supplemented with 100 μg/mL streptomycin, and incubated overnight under constant agitation at 37°C. 16 hours later, the culture was back-diluted 1:100 in 5 mL fresh TSB + 100 μg/mL streptomycin and incubated for another ~3 hours under constant shaking at 37°C. Upon reaching an OD_600_ of 1, bacteria were pelleted by centrifugation at 3,000×*g* for 8 minutes, washed twice with PBS, and resuspended in the same volume of pharmaceutical-grade PBS (USP) for infection by oral gavage. From several experiments that tracked the correlation between OD_600_ and colony-forming units (CFU), we can estimate that an OD_600_ equals roughly an infection dose of 1–2×10^8^ CFU/mL. Nevertheless, the actual infectious dose was determined for each experiment by serial dilution and plating on TSB agar plates containing 0.01% CR.

*Shigella flexneri* Δ*ipaH9.8* Strep^R^ was generated by transferring the K88R mutation of the *rpsL* gene from *Shigella flexneri* WT (streptomycin resistant)^103^ into the Δ*ipaH9.8* strain (Piro et al ^72^) using PCR and the λ red recombinase-mediated recombination system^104^. In short, the rpsL gene was amplified from the WT strain with PCR using Q5^®^ Hot Start High-Fidelity 2X Master Mix with forward (TTTACGCTGACCAATGACGC) and reverse (CGGCATCGCCCTAAAATTCG) primers following the manufacturer’s protocol and subsequent purification with the Monarch^®^ Spin PCR & DNA Cleanup Kit (NEB # T1130S). *Shigella flexneri* Δ*ipaH9.8* was transformed with the recombinase-carrying pKD46 (Datsenko et al.^104^) plasmid and selected on 0.01% CR TSB plates with 100μg/mL Ampicillin and 0.1% Glucose at 30°C. An overnight culture (TSB + Ampicillin + 0.1% Glucose and 30°C) was back-diluted 1:100 in 5mL TSB + Ampicillin and 0.1% L-Arabinose and incubated for another ~3 hours under constant shaking at 30°C. 1mL of bacteria were centrifuged at 13,000×*g* for 5min, washed three times with sterile H_2_O and resuspend in 200μL ice-cold H_2_O with 10% glycerol. 50μL of these competent cells were mixed with an oligonucleotide containing the point mutation and electroporated using a Gene Pulser Cuvette with 0.1cm gap size (BioRad #165–2089) and 1.8kV, 25μF (200Ω). Transformed bacteria were resuspended in 450μl SOC media (Thermo Fisher #15544034) and recovered at 37°C for 1h before being plated on CR TSB plates with 100μg/mL streptomycin.

#### *In vivo* infection

One day before infection, littermates or cohoused mice were deprived of food and water in the morning and 4–6 hours later orally gavaged with 100 μL of 250 mg/mL streptomycin sulfate dissolved in pharmaceutical-grade PBS, after which mice were again given food and water. On the day of infection, food and water were removed from the cage again, and 4–6 hours later, mice were orally gavaged with 100 μL of 10^8^ CFU/mL *S.f.,* prepared as described above. In compliance with our Animal Use Protocol, infected mice were visually examined twice per day and their change in weight was monitored every 24 hours. A loss of more than 25% of the initial weight measured on the day of infection (day 0), or signs of severe sickness such as pronounced hunching, combined with shivering or breathing distress, were considered humane endpoints at which mice were euthanized.

For *in vivo* antibody-mediated cytokine neutralization, 500 μg of anti-IFN-γ antibody (clone XMG1.2, Bio X Cell #BE055), anti-IL12 (clone C17.8, Bio X Cell #BE0051), anti-IL-18 (clone YIGIF74–1G7, Bio X Cell #BE0237), anti-IFNAR-1 (clone MAR1–5A3, Bio X Cell #BE0241) and InVivoPlus rat IgG1 isotype control (anti-HRP, Bio X Cell #BE0088) or InVivoPlus rat IgG2a isotype control (Bio X Cell #BE0089) depending on the isotype of the neutralizing antibody, were administered by daily intraperitoneal injection starting on the day of infection. For antibody-mediated depletion of neutrophils, 500μg of anti-Ly6G (clone 1A8, Bio X Cell #BE0075–1) was injected daily by intraperitoneal injection starting one day before infection (at the time of streptomycin administration).

In experiments involving diphtheria toxin-mediated cell-depletion, mice were injected i.p. with 30ng/g of DT (Sigma #D0564) or an equal volume of pharmacological PBS daily, starting one day before infection.

### METHOD DETAILS

#### Assessment of inflammation

If not indicated otherwise, 48 hours after infection, mice were sacrificed, their colon and cecum were isolated, and their lengths were recorded. Subsequently, the tissue was cut longitudinally, and the most distal fecal matter from the colon was collected, some of which was spread on detection tabs from a Hemoccult blood testing kit (Pro Advantage #P080018). The rest was then transferred into a pre-weighed 2 mL tube. The fecal matter was homogenized in 1 mL of PBS containing protease inhibitors (Roche #04693159001) using a polytron homogenizer. To determine the fecal CFU, serial dilutions were made in PBS and plated on TSB containing 0.01% CR and 100 μg/mL streptomycin sulfate. For lipocalin, calprotectin, and MPO ELISAs, samples were centrifuged at 13,000×*g* for 5 minutes and supernatants were analyzed in duplicates with R&D sandwich ELISA kits according to the manufacturer protocol.

A four-step scale was applied to quantify the presence of occult blood. A value of 0 indicates that the Hemoccult blood test was negative, 1 indicates a faint blue staining, and 2 indicates an intense staining. Mice that experienced severe hemorrhagic conditions with clearly visible, macroscopic blood in the lumen of the colon were assigned a score value of 3. Assessments were made on blinded samples.

#### IEC CFU

To determine the levels of intra-epithelial bacteria, the colon and cecum were isolated from infected mice 48 hours after infection as described above. After taking the fecal sample, the tissue was cleared of any remaining luminal content by washing in PBS. The tissue was collected in 5 mL RPMI (Thermo Fisher #21870–76) with 5% FBS, 2mM GlutaMax (Thermo Fisher #35050–61), 25mM HEPES (Thermo Fisher #15630–080), and 400 μg/mL gentamicin (Thermo Fisher #15710–064), and incubated for 1–2 hours at 4°C. Subsequently, tissue was washed six times in PBS, minced into approximately 1 cm pieces, and placed in 12 mL IEC stripping solution (HBSS, 25 HEPES, 2mM Glutamax, 50μg/mL Gentamicin, 2mM DTT, and 5mM EDTA) within a 50 mL Erlenmeyer flask and incubated for 30 minutes at 37°C and constant stirring at low speed (220rpm). The supernatant was passed through a 100 μm cell strainer, and the retained tissue was transferred back into the flask and mixed with 10 mL ice-cold PBS. The flask was sealed with a rubber stopping, shaken vigorously for 20 seconds, and combined with the previous extraction by passing through the same cell strainer. The remaining tissue was saved for further processing (see lamina propria ELISA or Flow staining). Next, the obtained IEC fraction was incubated with 50 μg/mL gentamicin on ice for 20 minutes, centrifuged 500×*g* for 5 minutes at 4°C, and washed twice with ice-cold PBS. Before the last centrifugation, a small aliquot of cells was taken for cell counting. Finally, the pellet was resuspended in 1 mL of 1% TritonX-100 (Fisher #BP151–100) and IEC-CFU was determined by plating serial dilutions on TSB agar plates with 0.01% CR and 100 μg/mL streptomycin sulfate.

#### Tissue ELISA

After extracting IEC cells, the remaining tissue was transferred into 14 ml round-bottom tubes containing 1 mL PBS with proteinase inhibitors (Roche #04693159001). After homogenization with a polytron homogenizer at 20,000 rpm, the suspension was centrifuged at 13,000×*g* for 5 minutes and the supernatant was analyzed in duplicates with R&D sandwich ELISA kits following the manufacturer protocol and normalized to the total protein concentration determined with the Pierce BCA protein assay kit (Thermo scientific #23225) according to the manufacturer’s protocol.

#### CFU Spleen/MLN

The spleen and mesenteric lymph nodes were isolated from infected mice 48 hours after infection. Collected tissue was placed in RPMI with 5% FBS, 2mM GlutaMax, 25mM HEPES, and 400 μg/mL gentamicin for 1h. After washing 5× with PBS, tissue was homogenized using a polytron homogenizer and serial dilutions plated on TSB agar plates with 0.01% CR and 100 μg/mL streptomycin sulfate.

#### IEC bead enrichment and RNAseq

The IEC faction was obtained as described above and further digested with Dispase-II to generate a single-cell suspension, as described by Gracz *et al*^105^. In brief, the pelleted IEC fraction was washed with 10 mL PBS with 10% PBS and resuspended in 10 mL of pre-warmed (37°C) HBSS containing 8 mg Dispase-II (Sigma #D4693). Tubes were incubated in a 37°C water bath for 10 minutes with vigorous shaking every 2 minutes. Next, 1 mL FBS with 500 μg DNase (Roche #11284932001) was added and incubated 3 minutes on ice. After washing with RPMI containing 10% FBS, cells were passed through a 40 μm cell strainer. Epithelial cells were enriched using the MojoSort^™^ Mouse CD326 (Ep-CAM) Selection Kit (BioLegend Cat #480141) following the manufacturer’s protocol for Positive Selection. Retained CD326^+^ cells were washed with 4mL RPMI containing 10% FBS and centrifuged at 1,000×*g* for 5 minutes at 4°C and lysed in 250 μL Trizol (Thermo Fisher #10296028). Samples were topped up with 100 μL DNase/RNase-free water and mixed with 200 μL chloroform (Fisher Scientific #C298–500). The aqueous phase and mixed with an equal volume of ethanol. Subsequently, the RNA was isolated using the Monarch Total RNA Miniprep Kit (NEB #T2010S) following the manufacturer’s protocol with on-column DNase treatment. The RNA obtained was sent to Azenta for quality assessment, automated PolyA selection, library preparation, multiplexing, and paired-end sequencing with a read length of 150 bases. After demultiplexing, raw data were pre-processed (including quality control as well as barcode, adaptor, and quality trimming) using FastQC (www.bioinformatics.babraham.ac.uk/projects/fastqc/) and cutadapt, mapped to the Mus_musculus.GRCm38.96 genome assembly using Kalisto (0.44.0 via Bioconda). Overall, each sample contained between 24 million and 32 million reads. Differential expression analysis was performed using DESeq2 (V1.44.0)^106^ with lfc shrinkage correction in R project version 4.4.1 with RStudio version 2024.04.2+764 (Foundation for Statistical Computing, Vienna, Austria, www.R-project.org/). For gene ontology (GO) enrichment analysis genes with differential expression values padj < 0.001 and log_2_ fold change > 1 were selected and enrichment analysis was performed using gseGO of the clusterProfiler (4.12.2) package for R with a gene set size <150.

#### Flow cytometry of lamina propria cells

To prepare cells from the lamina propria for flow cytometry, the colon and cecum were isolated from infected mice and stripped of IEC as described above. The collected remaining tissue was minced thoroughly with scissors and transferred into 50 mL Erlenmeyer flasks. After adding 10 mL of pre-warmed HBSS with 100 μg/mL Liberase TM (Sigma #5401127001) and 5 μg/mL DNase, the tissue was digested for 45 minutes under constant stirring at 37°C. The cell suspension was passed through a 70 μm strainer and washed twice with cold RPMI containing 10% FBS. Epithelial and dead cells were removed using a 70% and 40% two-phase Percoll gradient. Cells from the interface were subsequently collected, washed with PBS and stained with Ghost Dye^™^ Red 780 (Tonbo #SKU 13–0865-T100) before blocking the FcγII/III receptor with an anti-CD16/anti-CD32 antibody (BioLegend, #156604). Samples were stained for 45 minutes to an hour at room temperature in FACS buffer (PBS with 5g/L BSA and 2g/L sodium azid) with the following antibodies: BV785-labeled CD45 (Clone 104, BioLegend #109839), BUV496-labeled CD3 (Clone 145–2C11, BD #612955), APC-eFlour-labeled B220 (Clone RA3–6B2, Thermo Fisher #47–0452-82), PE-labeled CD11b (Clone M1/70 Thermo Fisher #12–0112-82), FITC-labeled Ly6G (Clone 1A8, BD #551460), APC-labeled Ly6C (Clone HK1.4, BioLegend #128016), MHCII (Colon M5/114.15.2, BioLegend #107626), BUV605-labeled CD64 (Clone H1.2F3, BioLegend #104530), BV421-labeled F4/80 (Clone BM8, BioLegend #123137) and APC-Cy7-labeled EpCAM (Clone G8.8, BioLegend #118218). Stained samples were washed twice and fixed with cytofix/cytoperm (BD biosciences #554722) for 10 minutes at room temperature before measured on an Aurora (Cytek) flow cytometer. Data were analyzed with Flowjo version 10 (BD Biosciences).

#### Blood Staining

Terminal blood sampling was performed by retro-orbital sinus puncture of anesthetized mice with a heparinized capillary (#22–260950 Fisher scientific). 15μL of the collected blood was immediately mixed with 100μL PBS containing 100U/mL heparin (#H0878–100KU Sigma) and 25μL counting beads (AccuCheck Counting Beads, Thermo Fisher #PCB100). After centrifugation and washing with FACS buffer, cell pellet was stained with 25μL of antibody mix (APC-labeled Ly6C (Clone HK1.4, BioLegend), FITC-labeled Ly6G (Clone 1A8, BD #551460), PE-labeled CD3 (ebio #12–0031-81 Clone 14–2011)) along with the viability dye (Tonbo #13–0658-T) and FcγII/III-block. Subsequently, cells were fixed cytofix/cytoperm (BD biosciences #554722) for 10 min and red blood cells were lysed with ACK buffer (Thermo fisher #A1049201). After washing twice with FACS buffer, samples were analyzed with an Aurora (Cytek) or a BD LSR Fortessa flow cytometer.

#### CT26 CRISPR-Cas9 Knockout and infection

CT26 were purchased from the Berkeley Cell Culture Facility and cultured in RPMI with 10% FBS, 2 mM Glutamax (Thermo Fisher #35050061), 10 mM HEPES (Thermo Fisher # 15630106), 1 mM sodium pyruvate (Thermo Fisher #11360070) and Penicillin-Streptomycin (Thermo Fisher #15070063). For gene disruption, CT26 cells were trypsinized, washed with media and electroporated with Cas9 2 NLS nuclease (Synthego) complexed with two gRNAs per gene (Synthego, sgRNA EZ kits), and Alt-R^®^ Cas9 Electroporation Enhancer (IDT, 1075916), in Lonza Cell Line Solution SE (Lonza, V4XC-10323) with buffer supplement 1 according to manufacturer’s protocol. Electroporation was performed with a Lonza 4D-Nucleofector Core Unit (AAF-1002B) using the program DS-120. Electroporated cells were immediately plated in media and knockout efficiency were determined by TIDE analysis of PCR products of the modified gnomic locus using Primestar PCR reagents according to manufacturer’s instructions and primers for Gbp1 (Fw: CAGCATTGGATGTTCTGCACTC and Rev: CTCATCATTTCCATAGGCTCACAC), Gbp2 (Fw: AGACTGTCAACATAGGAGGAACA and Rev: GGCTGATCCGCTGCTATTCT), Gbp5 (Fw: TGCTCCGGACAAGAAACTCC and Rev: CTTCACCAGAACTGCCTCGT) and Stat1 (Fw: TCTCGTTTGCGACCATCCG and Rev: ACCATCAGGGCCAGCATTAGG). All genotypes had a knockout efficiency of above 91%.The gRNA sequences used are the following (two per gene): Gbp1/2: CCTCAGTGTCAAGCAGAACC, GAGACTGCACAGTGGAGCCC, Gbp5: GCUUCCAACACUCAGCAACG, CUCAAACAUUCAAUCUACCG, Stat1: UUAAUGACGAGCUCGUGGAG, GGAUAGACGCCCAGCCACUG

For *in vitro* infections, 10^6^ CT26 cells were seeded into one well of a 6-well tissue culture treated plate in media without Penicillin-Streptomycin one day before the infection. *Shigella flexneri* - pFCcGi (constitutively expressing mCherry and an arabinose inducible GFP) was grown as described above. CT26 cells were pre-treated with 10 ng/mI IFN-γ (Peprotech #315–05-100UG) or left untreated for 16 hours and were spin-infected (600×*g* 10min at 37°C) with mid-log phase *Shigella* at an MOI of 1. After 45 minutes, cells were washed twice with PBS and medium exchanged to CT26 medium supplemented with 100 μg/ml gentamicin (Thermo fisher # 15710064) and 0.4% L-Arabinose (Sigma #A3256–25G). After 3 hours cells were isolated by trypsinization, washed, passed through a 70μm cell strainer, stained with Ghost Dye^™^ Red 780 (Tonbo #SKU 13–0865-T100), fixed with cytofix/cytoperm (BD biosciences #554722) for 10 min and analyzed with a BD LSR Fortessa flow cytometer.

#### Human organoid maintenance and infection

Deidentified human colonic organoids were a gift from Scott B. Snapper and established from a rectal biopsy obtained during a routine diagnostic endoscopy in a pediatric subject under Boston Children’s Hospital IRB protocol and cultured with methods modified from Sato *et al*.^107^. Briefly, organoids were maintained in 50μL Matrigel domes with human IntestiCult^™^ Organoid Growth Medium (STEMCELL #6010) supplemented with 10 μM of the ROCK1/ROCK2 inhibitor Y-27632 and Penicillin-Streptomycin. 16 hours before infection, organoids were stimulated with 10 ng/ml IFN-γ (Peprotech # 300–02-100UG). Before infection domes were washed with PBS and disrupted with a pipette tip and the Matrigel was digested with 0.25% Trypsin at 37°C for 5min. After washing with media, organoids were infected with mid-log phase *Shigella flexneri* - pFCcGi at an MOI of approximately 1. After 45 minutes, organoids were washed twice with PBS and medium exchanged to IntestiCult medium supplemented with 100 μg/ml gentamicin (Thermo fisher # 15710064) and 0.4% L-Arabinose (Sigma #A3256–25G). After 3 hours organoids were washed and, stained with Ghost Dye^™^ Red 780 (Tonbo #SKU 13–0865-T100). After single-cell digestion with TrypeLE (Thermo fisher #12604013) at 37°C for 5 minutes, cells were fixed with cytofix/cytoperm (BD biosciences #554722) for 10 min, passed through a cell strainer and analyzed with a BD LSR Fortessa flow cytometer.

### QUANTIFICATION AND STATISTICAL ANALYSIS

Data were analyzed using GraphPad Prism10 software and as indicated in the figure legends. Statistical parameters are reported in the figure legends and supplementary material. Statistical tests employed include unpaired Student’s t-test, Mantel-Cox test, 1-way or 2-way ANOVA with Tukey’s multiple comparison or Kruskal-Wallis test with Dunn’s corrections, to obtain the indicated p-values with ****p<0.0001, ***p<0.001, **p<0.01, ****p<0.05, ns = not significant (p>0.05).

## Supplementary Material

1

2 Supplemental Videos and Spreadsheets_Table1: Differential expression IEC naïve vs infected, related to Figure 1c.

3 Supplemental Videos and Spreadsheets_Table2: Differential expression of ISGs in IEC naïve vs infected, related to Figure 1c.

## Figures and Tables

**Key resources table:** 

REAGENT or RESOURCE	SOURCE	IDENTIFIER
Antibodies		
anti-CD16/anti-CD32 antibody	BioLegend	Cat# 156604; RRID:AB_2783138
APC CD11c	BioLegend	Cat# 117310; RRID:AB_313779
APC CD19	BioLegend	Cat# 152409; RRID:AB_2629838
APC CD3	Thermo Fisher Scientific	Cat# 17-0031-82; RRID:AB_469315
APC CD5	BioLegend	Cat# 100625; RRID:AB_2563928
APC F4/80	Thermo Fisher Scientific	Cat# 17-4801-80; RRID:AB_2784647
APC Ly6C	BioLegend	Cat# 128016; RRID:AB_1732076
APC Ly6G	BioLegend	Cat# 127614; RRID:AB_2227348
APC NKp46	BioLegend	Cat# 137608; RRID:AB_10612758
APC-Cy7 EpCAM	BioLegend	Cat# 118218 RRID:AB_2098648
APC-Cy7 MHCII	BioLegend	Cat# 107627; RRID:AB_1659252
APC-eFlour780^™^ CD45R	Thermo Fisher Scientific	Cat# 47-0452-82; RRID:AB_1518810
APC-eFlour780^™^ KLRG1	Thermo Fisher Scientific	Cat# 100625; RRID:AB_2573987
BUV395 CD45	BD	Cat# 749889; RRID:AB_2874129
BUV496 CD3	BD	Cat# 612955 RRID:AB_2870231
BUV496 TCRb	BD	Cat# 749915; RRID:AB_2874154
BV421 CX3CR1	BioLegend	Cat# 149023; RRID:AB_2565706
BV421 F4/80	BioLegend	Cat# 123137; RRID:AB_2563102
BV421 NKp46	BioLegend	Cat# 137612; RRID:AB_2563104
BV605 CD127	BioLegend	Cat# 135041; RRID:AB_2572047
BV605 CD64	BioLegend	Cat# 104530; RRID:AB_2629778
BV605 EpCAM	BioLegend	Cat# 118227; RRID:AB_2563984
BV785 CD45	BioLegend	Cat# 109839; RRID:AB_2562604
cytofix/cytoperm	BD	Cat# 554722; RRID:AB_2869010
FITC CD11c	Thermo Fisher Scientific	Cat# 11-0114-82; RRID:AB_464940
FITC Ly6G	BD	Cat# 551460; RRID:AB_394207
Ghost Dye^™^ Red 780	Tonbo	Cat# SKU 13-0865-T100
Ghost Dye^™^ Violet 540	Tonbo	Cat# SKU 13-0879-T100
PE CD11b	Thermo Fisher Scientific	Cat# 12-0112-82; RRID:AB_2734869
PE CD64	BioLegend	Cat# 139304; RRID:AB_10612740
PE Eomes	BioLegend	Cat# 157705; RRID:AB_2888891
PE-Cy5 CD11b	BioLegend	Cat# 101209; RRID:AB_312792
PE-Cy7 EpCAM	BioLegend	Cat# 118216; RRID:AB_1236471
PerCP-Cy5.5 Ly6C	Thermo Fisher Scientific	Cat# 45-5932-80; RRID:AB_2723342
PerCP-Cy5.5 MHCII	BioLegend	Cat# 107626; RRID:AB_2191071
PerCP-Cy5.5 TCRgd	BioLegend	Cat# 118117; RRID:AB_10612572
R718 RORyt	BD	Cat# 567362; RRID:AB_2916571
InVivoPlus anti-mouse IFNγ	Bio X Cell	Cat# BE0055; RRID:AB_1107694
InVivoPlus rat IgG1 isotype control (anti-HRP)	Bio X Cell	Cat# BE0088; RRID:AB_1107775
InVivoPlus anti-mouse IL-12 p40	Bio X Cell	Cat# BE0051; RRID:AB_1107698
InVivoMab anti-mouse IL-18	Bio X Cell	Cat# BE0237; RRID:AB_2687719
InVivoPlus anti-mouse Ly6G	Bio X Cell	Cat# BE0075-1; RRID:AB_1107721
InVivoMAb anti-mouse IFNAR-1	Bio X Cell	Cat# BE0241, RRID:AB_2687723
InVivoPlus rat IgG2a isotype control	Bio X Cell	Cat# BE0089; RRID:AB_1107769
MojoSort^™^ Mouse CD326 (Ep-CAM) selection kit	BioLegend	Cat# 480142
AccuCheck Counting Beads	Thermo Fisher Scientific	Cat# PCB100
		
Bacterial and virus strains		
*Shigella flexneri*: serovar 2a 2457T strain, natural streptomycin-resistant	Mitchell et al.^15^	N/A
*Shigella flexneri*: serovar 2a 2457T strain *ΔipaH9.8*	Lesser Lab Piro et al.^72^	N/A
*Shigella flexneri*: serovar 2a 2457T strain *ΔipaH9.8*, streptomycin-resistant	This study	N/A
*Shigella flexneri*: serovar 2a 2457T strain Virulence-plasmid cured	Lesser Lab Lampel et al.^5^	N/A
		
Biological samples		
Deidentified human colonic organoids	Scott B. Snapper (see acknowledgments)	N/A
		
Chemicals, peptides, and recombinant proteins		
Diphtheria toxin	Sigma-Aldrich	Cat# D0564
cOmplete^™^, Mini, EDTA-free Protease Inhibitor Cocktail	Sigma-Aldrich	Cat# 04693159001
Mouse IFN-gamma Quantikine ELISA Kit	R&D Systems	Cat# DY485-05
Mouse S100A8/S100A9 Heterodimer DuoSet ELISA	R&D Systems	Cat# DY8596-05
Mouse Lipocalin-2/NGAL DuoSet ELISA	R&D Systems	Cat# DY1857-05
Mouse Myeloperoxidase DuoSet ELISA	R&D Systems	Cat# DY3667
Mouse IL-1 beta/IL-1F2 DuoSet ELISA	R&D Systems	Cat# DY401-05
Mouse CXCL1/KC DuoSet ELISA	R&D Systems	Cat# DY453-05
Dispase-II	Sigma-Aldrich	Cat# D4693
DNase	Sigma-Aldrich	Cat# 11284932001
Liberase^™^	Sigma-Aldrich	Cat# 5401127001
Heparin	Sigma-Aldrich	Cat# #H0878-100KU
ACK buffer	Thermo Fisher Scientific	Cat# A1049201
SpCas9 2 NLS nuclease	Synthego	
Alt-R^®^ Cas9 Electroporation Enhancer	IDT	Cat# 1075916
Recombinant mouse IFN-γ	Peprotech	Cat# 315-05-100UG
Recombinant human IFN-γ	Peprotech	Cat# 300-02-100UG
IntestiCult^™^ Organoid Growth Medium	StemCell	Cat# 6010
Y-27632	StemCell	Cat# 72304
		
Critical commercial assays		
Hemoccult blood testing kit	Pro Advantage	Cat# P080018
Pierce BCA protein assay kit	Thermo Fisher Scientific	Cat# 23225
Monarch Total RNA Miniprep Kit	NEB	Cat# T2010S
Lonza Cell Line Solution SE	Lonza	V4XC-10323
		
Deposited data		
Raw and analyzed RNA-Seq data from EpCAM enriched cecum/colon cells	This paper	GEO: GSE288567
		
Experimental models: Cell lines		
Mouse: CT26	Berkeley Cell Culture Facility	RRID:CVCL_7254
		
Experimental models: Organisms/strains		
Mouse: *Nlrc4^−/−^*: C57BL/6J-Nlrc4^542stop^/J	Tenthorey et al.^96^	Jax# 039847 (about to be publicly released from Jax)
Mouse: *Nlrc4^−/−^Casp11^−/−^*: C57BL/6J- Nlrc4^542stop^ Casp4^em13JLR^/J	Roncaioli et al.^16^	N/A
Mouse: *Lyz2^Cre^*: B6.129P2-Lyz2^tm1(cre)Ifo^/J	The Jackson Laboratory	RRID:IMSR_JAX:004781
Mouse: *Mrp8^Cre^*: B6.Cg-Tg^(S100A8-cre,-EGFP)1Ilw^/J	The Jackson Laboratory	RRID:IMSR_JAX:021614
Mouse: *CD64^Cre^*: B6-Fcgr1^tm2Ciphe^	Bernard Malissen Scott et al.^50^	N/A
Mouse: *iDTR^lsl/lsl^*: C57BL/6-Gt(ROSA)26Sor^tm1(HBEGF)Awai^/J	The Jackson Laboratory	RRID:IMSR_JAX:007900
Mouse: *Ifngr1^−/−^*: B6.129S7-Ifngr1^tm1Agt^/J	The Jackson Laboratory	RRID:IMSR_JAX:003288
Mouse: *Tlr2^−/−^Tlr4^−/−^Unc93b1^−/−^* (C57BL/6N-*Unc93b1^tm1(KOMP)Vlcg^*/Mmucd)	Barton Lab Sivick et al.^52^	N/A
Mouse: *Il12^−/−^*: B6.129S1-Il12^btm1Jm^/J	The Jackson Laboratory	RRID:IMSR_JAX:002693
Mouse: *Zbtb46^DTR/DTR^*: B6(Cg)-*Zbtb46*^tm1(HBEGF)Mnz^/J	The Jackson Laboratory	RRID:IMSR_JAX:019506
Mouse: GREAT reporter: B6.129S4-Ifng^tm3.1Lky^/J	The Jackson Laboratory	RRID:IMSR_JAX:017581
		
Oligonucleotides		
sgRNA targeting sequence: Gbp1 & Gbp2 #1: CCTCAGTGTCAAGCAGAACC	This study Ordered form Synthego	N/A
sgRNA targeting sequence: Gbp1 & Gbp2 #2: GAGACTGCACAGTGGAGCCC	This study Ordered form Synthego	N/A
sgRNA targeting sequence: Gbp5 #1: GCUUCCAACACUCAGCAACG	This study Ordered form Synthego	N/A
sgRNA targeting sequence: Gbp5 #2: CUCAAACAUUCAAUCUACCG	This study Ordered form Synthego	N/A
sgRNA targeting sequence: Stat1 #2: UUAAUGACGAGCUCGUGGAG	This study Ordered form Synthego	N/A
sgRNA targeting sequence: Stat1 #2: GGAUAGACGCCCAGCCACUG	This study Ordered form Synthego	N/A
		
Recombinant DNA		
p46 (carries the araBAD promoter driven λ red recombinase genes)	Lesser Lab Datsenko et al.^104^	N/A
pFPV-mCherry	Olivia Steele-Mortimer^109^	RRID:Addgene_20956
pFCcGi	Sophie Helaine & David Holden^108^	RRID:Addgene_59324
		
Software and algorithms		
FastQC	Andrews, S	www.bioinformatics.babraham.ac.uk/projects/fastqc/
cutadapt	Martin, M	https://cutadapt.readthedocs.io/en/stable/
Kallisto	Nicolas L Bray	https://pachterlab.github.io/kallisto/about
DESeq2	Love et al.^106^	https://bioconductor.org/packages/release/bioc/html/DESeq2.html
R	Version 4.4.1	https://cran.rstudio.com/
clusterProfiler	Version 4.12.2	https://bioconductor.org/packages/devel/bioc/html/clusterProfiler.html
GraphPad Prism 9	GraphPad Software	https://www.graphpad.com/
BioRender	BioRender Company	https://BioRender.com/64uv53d https://BioRender.com/1dzqp93
		
Other		
		

## Highlights

- Neutrophils are dispensable for *Shigella* control in vivo.
- Macrophages sense *Shigella* via TLRs and produce IL-12.
- IL-12 induces IFN-γ, essential for controlling *Shigella* replication.
- IFN-γ limits *Shigella* in intestinal epithelial cells to resolve infection in vivo.
