# Supplementary material for: Macrophages orchestrate elimination of Shigella from the intestinal epithelial cell niche via TLR-induced IL-12 and IFN-γ
Source: Cell Host Microbe. Author manuscript; Available in PMC 2025 Sep 26. (PMC12471108; doi:10.1016/j.chom.2025.08.001)
Supplement: 1 [file NIHMS2107783-supplement-1.pdf]

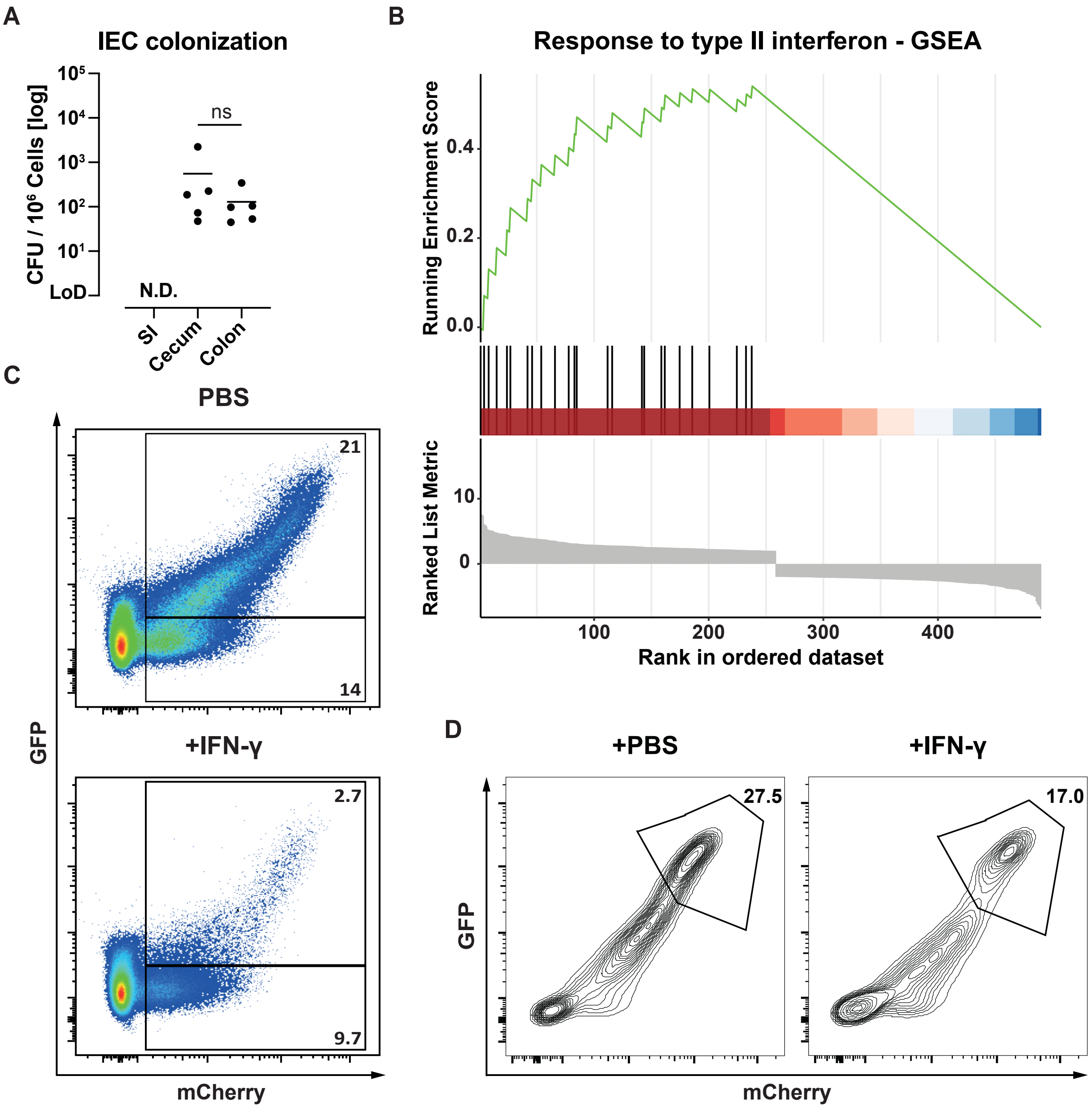

Figure S1: *Shigella* exclusively colonizes the cecum and colon, response to type II interferon limits its intracellular replication *in vitro*, related to Figure 1.

(A) Intracellular CFU per million epithelial cells isolated from the small intestine (SI), cecum, and colon, 48h after infection. **(B)** Gene set enrichment analysis for the biological process "Responses to type II interferon" identified in Fig. 1D plotted as running enrichment scores (ES) and positions of gene set members on the rank-ordered list of the differently expressed genes. **(C**) Representative plots for quantifying infected ($GFP^{+}mCherry^{+}$) CT26 cells shown in Fig. 1E and **(D)** from human colonic organoids shown in Fig. 1F**.** (A) n=5, median, Mann-Whitney-test, LoD=Limit of detection, N.D.=no CFUs detected.

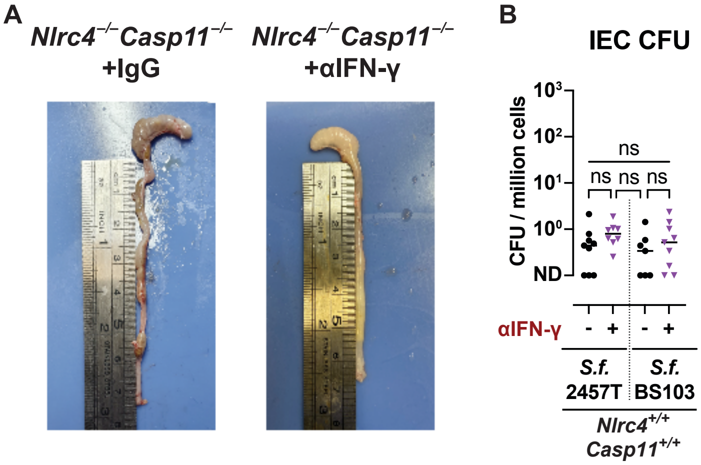

Figure S2: IFN-γ neutralization enhances atrophy but not bacterial burdens in WT mice, related to Figure 2.

(A) Representative images of cecum and colon isolated from *Nlrc4*$^{-/-}$*Casp11*$^{-/-}$ mice 48 hours after oral infection with *Shigella* and simultaneous treatment with IFN-γ-neutralizing antibody (αIFN-γ, right) or isotype control (IgG, left). **(B)** Effect of IFN-γ-neutralization on wild-type C56BL/6J mice assessed by treatment with anti-IFN-γ antibody or isotype control and infection with virulent *S.f.* 2457T or the avirulent strain BS103. ND=no colonies detected. (B) n=8-9, median and Kruskal-Wallis test with Dunn's multiple comparison.

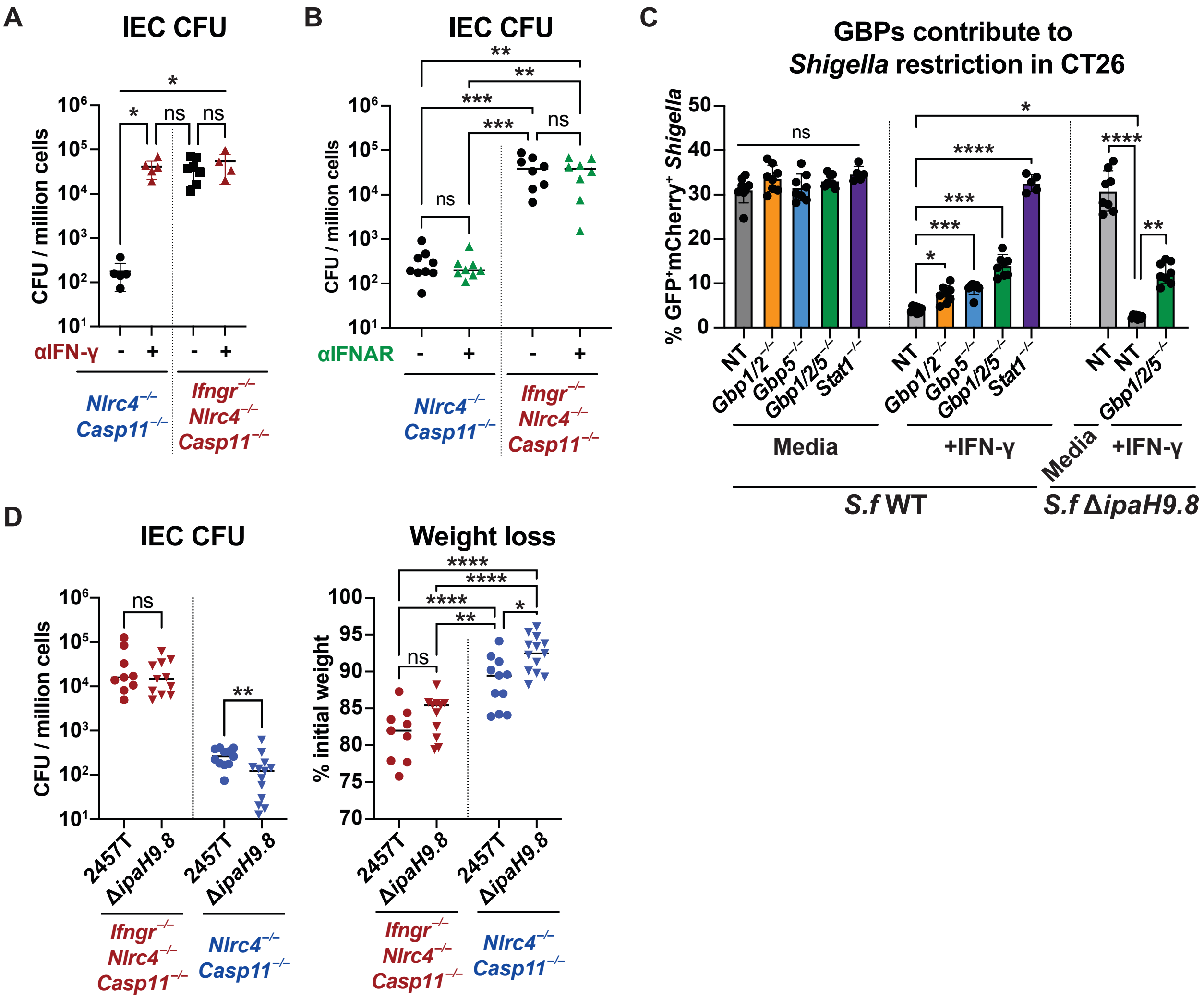

Figure S3: IFN-γ-mediated protection is partially GBP-dependent with no role for type I IFN, related to Figure 3.

(**A**) IEC CFUs from infected *Ifngr1*$^{-/-}$*Nlrc4*$^{-/-}$*Casp11*$^{-/-}$ or *Nlrc4*$^{-/-}$*Casp11*$^{-/-}$ mice treated or not treated with IFN-γ neutralizing antibody. (**B**) comparison of IEC CFU counts from infected *Ifngr*$^{-/-}$ *Nlrc4*$^{-/-}$*Casp11*$^{-/-}$ or *Nlrc4*$^{-/-}$*Casp11*$^{-/-}$ treated with IFNAR blocking antibody (+) or an isotype control (-). **(C)** CT26 cells with CRISPR-Cas9 knockout of *Gbp1/2*, *Gbp5*, *GBP1/2/5*, or *Stat1*, or non-targeting (NT) scramble gRNA controls, were infected with WT *Shigella* or an *IpaH9.8*-deficient strain, both expressing mCherry and arabinose-inducible GFP. **(D)** Intra-epithelial CFU and change in body weight of *Ifngr1*$^{-/-}$*Nlrc4*$^{-/-}$*Casp11*$^{-/-}$ or *Nlrc4*$^{-/-}$*Casp11*$^{-/-}$ mice infected with either WT or an *IpH9.8*-deficient strain. n=4-5/group (A), n=7-8/group (B), n=5-8/condition (C) and n=9-13 (D). Mean, SD and one-way ANOVA with Tukey's multiple comparison of indicated samples in (C), weight loss in (D), median and Kruskal-Wallis test with Dunn's multiple comparison for CFU in (A,C and D).

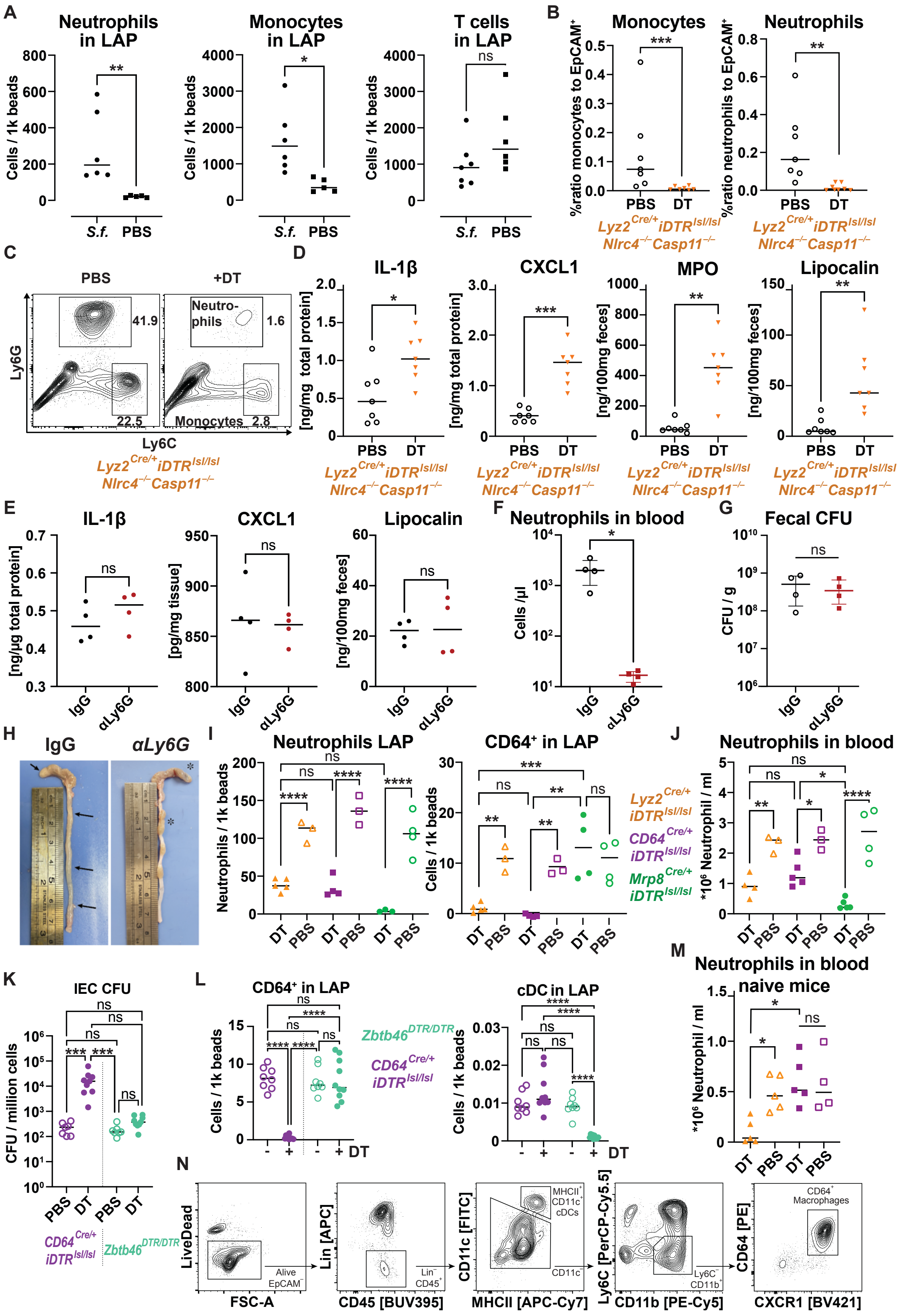

A
Neutrophils in LAP
Monocytes in LAP
T cells in LAP
Cells / 1k beads
S.f.
PBS
B
Monocytes
Neutrophils
%ratio monocytes to EpCAM+
%ratio neutrophils to EpCAM+
PBS
DT
Lyz2Cre/+ iDTRlsl/lsl Nlrc4−/−Casp11−/−
C
PBS
+DT
Neutro-phils
Monocytes
Ly6G
Ly6C
D
IL-1β
CXCL1
MPO
Lipocalin
[ng/mg total protein]
[ng/100mg feces]
E
[ng/µg total protein]
[pg/mg tissue]
IgG
αLy6G
F
Neutrophils in blood
Cells /µl
G
Fecal CFU
CFU / g
H
I
Neutrophils LAP
CD64+ in LAP
Neutrophils / 1k beads
Lyz2Cre/+ iDTRlsl/lsl
CD64Cre/+ iDTRlsl/lsl
Mrp8Cre/+ iDTRlsl/lsl
J
Neutrophils in blood
*10^6 Neutrophil / ml
K
IEC CFU
CFU / million cells
Zbtb46DTR/DTR
L
cDC in LAP
M
Neutrophils in blood naive mice
N
LiveDead
FSC-A
Alive EpCAM−
Lin [APC]
CD45 [BUV395]
Lin− CD45+
CD11c [FITC]
MHCII [APC-Cy7]
MHCII+ CD11c+ cDCs
CD11c−
Ly6C [PerCP-Cy5.5]
CD11b [PE-Cy5]
Ly6C− CD11b+
CD64 [PE]
CXCR1 [BV421]
CD64+ Macrophages

Figure S4: Increased bowel inflammation in myeloid-deficient mice, related to Figure 4.

(A) Neutrophils, monocytes and T cells in the lamina propria (LAP) of infected (*S.f.*) or naïve (PBS) *Nlrc4*$^{-/-}$*Casp11*$^{-/-}$ mice. **(B)** Monocyte and neutrophil in the colon/cecum of infected *Lyz2*$^{Cre/+}$*iDTR*$^{lsl/lsl}$*Nlrc4*$^{-/-}$*Casp11*$^{-/-}$ mice with or without DT **(C)** representative flow plots of the data in B. **(D)** Effect of myeloid cell-depletion on IL-1β, CXCL1 protein levels in the LAP, and MPO and Lipocalin in the feces. **(E)** Impact of neutrophil depletion (anti-Ly6G) on the levels of IL-1β and CXCL1 in the LAP, and lipocalin in the feces, of infected *Nlrc4*$^{-/-}$*Casp11*$^{-/-}$ mice. **(F)** Quantification of circulating neutrophils in the blood and **(G)** luminal colonization of infected animals treated with anti-Ly6G or an isotype control. **(H)** Representative images of the cecum and colon of infected *Nlrc4*$^{-/-}$*Casp11*$^{-/-}$ mice treated with anti-Ly6G or an isotype. Arrows indicate sticky, white-to-yellow pus observed in infected *Nlrc4*$^{-/-}$*Casp11*$^{-/-}$ (left) that is absent upon antibody-mediated neutrophil depletion (right). **(I)** Ly6G$^+$ neutrophils and CD64$^+$ macrophages in the LAP and (J) blood from infected, irradiated *Nlrc4*$^{-/-}$*Casp11*$^{-/-}$ mice, engrafted with *Lyz2*$^{Cre/}$ *iDTR*$^{lsl/lsl}$, *CD64*$^{Cre/+}$*iDTR*$^{lsl/lsl}$, or *MRP8*$^{Cre/+}$*iDTR*$^{lsl/lsl}$ BM, and treated with DT or PBS (n=3-4 per group). **(M)** Number of Ly6G$^+$ neutrophils before infection. **(K)** IEC CFU counts from irradiated *Nlrc4*$^{-/-}$*Casp11*$^{-/-}$ mice receiving *CD64*$^{Cre/+}$*iDTR*$^{lsl/lsl}$ or *Zbtb46*$^{DTR/DTR}$ BM and treated with DT or PBS during the course of infection (n=7-10 per group). **(L)** (top) Quantification of CD64$^+$ (Alive EpCAM$^-$CD45$^+$Lin$^-$CD11c$^-$CD11b$^+$Ly6C$^-$CD64$^+$) and conventional dendritic cells (Alive EpCAM$^-$CD45$^+$Lin$^-$CD11c$^+$MHCII$^+$) in the LAP of mice from (K). **(N)** Gating strategy for CD64$^+$ macrophages and cDC. Cells were pre-gated for EpCAM$^-$ cells isolated from infected mice and using Ly6G, CD3, CD5, Nkp46, and CD19 as lineage markers.

n=5-6 in A, n=7/group in B and D, n=4 in E-G, and n=3-5 in I-J. Median and mann-Whitney-test for (A,B,D,F,G), mean and t-test for (E), mean and ANOVA with Dunnett's multiple comparisons test in (I,J,L,M), median and Kruskal-Wallis test with Dunn's multiple comparison in (K).

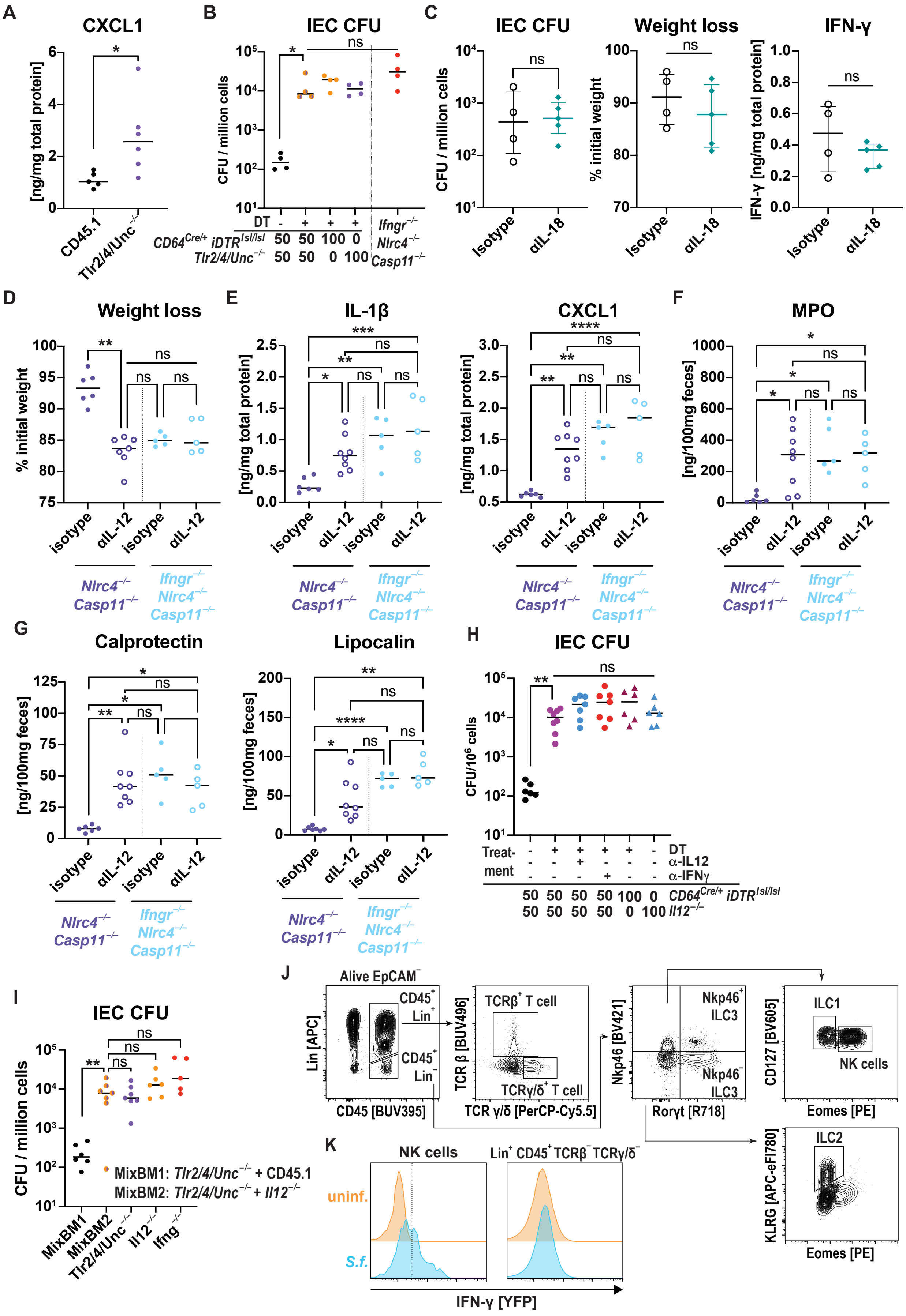
A
CXCL1
[ng/mg total protein]
CD45.1
Tlr2/4/Unc−/−
B
IEC CFU
CFU / million cells
DT
CD64Cre/+ iDTRlsl/lsl
Tlr2/4/Unc−/−
Ifngr−/−
Nlrc4−/−
Casp11−/−
C
IEC CFU
CFU / million cells
Weight loss
% initial weight
IFN-γ
IFN-γ [ng/mg total protein]
Isotype
αIL-18
D
Weight loss
% initial weight
E
IL-1β
[ng/mg total protein]
CXCL1
[ng/mg total protein]
F
MPO
[ng/100mg feces]
isotype
αIL-12
Nlrc4−/−
Casp11−/−
Ifngr−/−
Nlrc4−/−
Casp11−/−
G
Calprotectin
[ng/100mg feces]
Lipocalin
[ng/100mg feces]
H
IEC CFU
CFU/10⁶ cells
Treat-ment
DT
α-IL12
α-IFNγ
CD64Cre/+ iDTRlsl/lsl
Il12−/−
I
IEC CFU
CFU / million cells
MixBM1: Tlr2/4/Unc−/− + CD45.1
MixBM2: Tlr2/4/Unc−/− + Il12−/−
MixBM1
MixBM2
Tlr2/4/Unc−/−
Il12−/−
Ifng−/−
J
Alive EpCAM−
CD45+ Lin+
CD45+ Lin−
Lin [APC]
CD45 [BUV395]
TCRβ+ T cell
TCRγ/δ+ T cell
TCR β [BUV496]
TCR γ/δ [PerCP-Cy5.5]
Nkp46+ ILC3
Nkp46− ILC3
Nkp46 [BV421]
Rorγt [R718]
ILC1
NK cells
CD127 [BV605]
Eomes [PE]
ILC2
KLRG [APC-eFl780]
Eomes [PE]
K
NK cells
Lin+ CD45+ TCRβ− TCRγ/δ−
uninf.
S.f.
IFN-γ [YFP]

Figure S5: IL-12p40 neutralization phenocopies IFN-γR deficiency, whereas IL-18 neutralization has no effect, related to Figure 5.

(A) ELISA for CXCL1 in the lamina propria (LAP) 48 hours post-infection in irradiated *Nlrc4*$^{-/-}$ *Casp11*$^{-/-}$, receiving *Tlr2/4-Unc*$^{-/-}$ or CD45.1-WT BM. **(B)** IEC CFU counts obtained from irradiated *Nlrc4*$^{-/-}$*Casp11*$^{-/-}$ receiving either a 50%:50% mix or 100% of BM from *CD64*$^{Cre/+}$*iDTR*$^{lsl/lsl}$ and/or *Tlr2/4/Unc*$^{-/-}$. DT (+) or PBS(-) treatment from day -1 on throughout the infection. **(C)** IEC CFU, weight loss and IFN-γ levels in the LAP of *Nlrc4*$^{-/-}$*Casp11*$^{-/-}$ treated with an IL-18 neutralizing antibody or an isotype control. **(D)** % initial weight, **(E)** IL-1β and CXCL1 levels in LAP, (**F)** MPO, **(G)** Calprotectin and Lipocalin in the feces of infected *Nlrc4*$^{-/-}$*Casp11*$^{-/-}$ or *Ifngr*$^{-/-}$*Nlrc4*$^{-/-}$*Casp11*$^{-/-}$ additionally treated with αIL-12 or isotype control. **(H)** IEC CFU counts of irradiated *Nlrc4*$^{-/-}$*Casp11*$^{-/-}$ receiving either a 50%:50% mix or 100% of BM from *CD64*$^{Cre/+}$*iDTR*$^{lsl/lsl}$ and/or *Il12*$^{-/-}$ and additionally treated with DT, anti-IL12p40 and anti-IFN-γ throughout the infection. **(I)** IEC CFU from irradiated *Nlrc4*$^{-/-}$*Casp11*$^{-/-}$ engrafted with either a mix of *Tlr2/4/Unc*$^{-/-}$ and CD45.1, a mix of *Tlr2/4/Unc*$^{-/-}$ and *Il12b*$^{-/-}$, *Tlr2/4/Unc*$^{-/-}$, *Il12b*$^{-/-}$ or *Ifng*$^{-/-}$ BM. **(J)** Gating strategy for Fig. 5H, pre-gating for alive and EpCAM^{-}, and staining for CD3, CD5^{+}, CD19^{+}, Ly6G^{+}, Ly6C^{+}, MHCII^{+}, F4/80^{+} as linage markers (top). **(K)** Representative histogram. Mean and t-test (A,C), ANOVA with Tukey's multiple comparisons (D,E,F,G), or median and Dunn's multiple comparisons test (B,H,I). n=5 (A), n=4-5 (C), n=5-8 (D-G), n=5-8 (H,I)
